# Development of a Lipid-encapsulated TGFβRI-siRNA Drug for Liver Fibrosis Induced by *Schistosoma mansoni*

**DOI:** 10.1371/journal.pntd.0012502

**Published:** 2024-09-12

**Authors:** Ying-Chou Chen, Yueh-Lun Lee, Ching-An Lee, Tzu-Yuan Lin, Edwin En-Te Hwu, Po-Ching Cheng

**Affiliations:** 1 Graduate Institute of Medical Sciences, College of Medicine, Taipei Medical University, Taipei, Taiwan; 2 Department of Molecular Parasitology and Tropical Diseases, School of Medicine, College of Medicine, Taipei Medical University, Taipei, Taiwan; 3 Drug Metabolism & Pharmacokinetics Department, Institute for Drug Evaluation Platform, Development Center for Biotechnology, Taipei, Taiwan; 4 Department of Microbiology and Immunology, School of Medicine, College of Medicine, Taipei Medical University, Taipei, Taiwan, Taiwan; 5 The Danish National Research Foundation and Villum Foundation’s Center for Intelligent Drug Delivery and Sensing Using Microcontainers and Nanomechanics, Department of Health Technology, Technical University of Denmark; 6 Center for International Tropical Medicine, College of Medicine, Taipei Medical University, Taipei, Taiwan; Seoul National University College of Medicine, REPUBLIC OF KOREA

## Abstract

*Schistosoma mansoni* infection leads to chronic schistosomiasis and severe hepatic fibrosis. We designed a liver-targeted lipid nanoparticle (LNP) carrying siRNA against type I TGF-β receptor (TGFβRI) mRNA to treat schistosomiasis-induced liver fibrosis in BALB/c mice. Knockdown of TGFβRI by LNP-siTGFβRI reduced LX-2 cell activation *in vitro* and alleviated liver fibrosis in *S*. *mansoni*-infected mice. *αSMA* and *Col1a1* fibrotic markers in the liver tissues of infected mice were significantly suppressed in the treatment groups. In the serum of the LNP-siTGFβRI-treated groups, cytokines IFNγ, IL-1α, IL-6, IL-12, RANTES (CCL5), and TNFα increased, while GM-CSF, IL-2, IL-4, IL-10, IL-13, and KC (CXCL1) decreased compared to the control. Cell proportions were significantly altered in *S*. *mansoni*-infected mice, with increased CD56d NK cells and decreased CD19^+^ B cells and CD4^+^ T cells compared to naïve mice. Following LNP-siTGFβRI treatment, CD56d NK cells were downregulated, while B and memory Th cell populations were upregulated. The density of fibrotic regions significantly decreased with LNP-siTGFβRI treatment in a dose-dependent manner, and no systemic toxicity was observed in the major organs. This targeted siRNA delivery strategy effectively reduced granulomatous lesions in schistosomiasis-induced liver fibrosis without detectable side effects.

## Author summary

This present study indicates that a liver-targeted lipid nanoparticles (LNPs) carrying siRNA against type I TGF-β receptor (TGFβRI) mRNA that we designed could be used to treat schistosomiasis-induced liver fibrosis. We demonstrated a liver-specific accumulation of LNPs upon intravenous injection in BALB/c mice at drug dose of 0.1 or 1 mg/kg twice a week for 4 weeks. In vitro and in vivo experiments both revealed reduced activation of HSC and alleviated liver fibrosis in S. mansoni-infected mice. The fibrotic markers αSMA and Col1a1 and serum cytokines including GM-CSF, IL-2, IL-4, IL-10, IL-13 and KC (CXCL1) from liver tissues were significantly suppressed in LNP-siTGFβRI treatment groups. FACS results indicated that following LNP-siTGFβRI treatment, CD56d NK cells showed downregulation, while B cell and memory Th cell populations were upregulated. Histological analysis showed the density of fibrotic regions were significantly decreased with LNP-siTGFβRI treatment in a dose-dependent manner and no systemic toxicity was observed in major organs. In this study, we provide a potentially targeted siRNA delivery strategy reducing granulomatous lesions for schistosomiasis-induced liver fibrosis treatment without detectable side effects. It is hoped that this study will provide effective new treatments for liver fibrosis caused by schistosomiasis in the future.

## 1 Introduction

Schistosomiasis is a neglected disease prevalent in tropical and subtropical regions. According to the WHO, approximately 250 million people globally suffer from *Schistosoma* infections, which present various pathological characteristics [1,2]. The primary species affecting humans are *Schistosoma mansoni*, *Schistosoma japonicum*, and *Schistosoma haematobium*. These infections are endemic in the Philippines, Indonesia, China, the Middle East, various nations of Africa, and several South America countries [3–5]. Infection occurs when cercariae (young worms) penetrate the skin and enter the bloodstream. After about five weeks, adult male and female worms mate and begin producing eggs. *S*. *haematobium* resides in the venous plexus causes urogenital schistosomiasis, while *S*. *mansoni* and *S*. *japonicum* inhabit the mesenteric veins, leading to hepatosplenic and intestinal schistosomiasis.

The pathology of *S*. *mansoni* infection includes acute and chronic phases [6,7]. In the early acute phase (3–5 weeks post-infection), type 1 helper T cells (Th1) mediate the immune response by secreting cytokines such as interferon gamma (IFNγ), interleukin 1 (IL-1), and tumor necrosis factor-alpha (TNFα), targeting the migrating schistosomula. In the chronic phase (6–10 weeks post-infection), eggs trapped in the liver secrete soluble egg antigens (SEAs) that attract macrophages, lymphocytes, eosinophils, and hepatic stellate cells (HSCs), leading to granuloma formation [8–10]. SEAs provoke a switch from a Th1 to a Th2-mediated immunity response, ultimately causing liver fibrosis [11–13]. Th2 cells secrete IL-4, IL-10, and IL-13, which activate alternatively activated macrophages (AAMφ) to release transforming growth factor-beta (TGF-β), inducing HSC activation. HSCs, which comprise 5–8% of liver cells [14], play critical roles in defending against *S*. *mansoni*-induced inflammation and liver fibrosis. Normally, quiescent HSCs in the space of Disse store vitamin A. TGF-β binds to TGF-β receptors (TGFβRs) on HSCs, activating the TGF-β/SMAD signaling pathway and leading to the phosphorylation of Samd2, Smad3, and Smad4, which regulate alpha-smooth muscle actin (αSMA) expression [14–16]. Activated HSCs transform into myofibroblasts, producing extracellular matrix (ECM) components like collagen to encompass granulomatous lesions [17–20]. Granulomatous responses, while protective, often lead to pathological fibrosis and irreversible organ damage. Praziquantel can eliminate mature schistosomes but does not address the pathological injury caused by schistosome eggs [8,9,18,21]. Effective therapeutic methods for targeted delivery and reversal of schistosomiasis-induced liver fibrosis are lacking [22].

*Schistosoma* produces various molecules affecting host gene expression and cell recognition, limiting the host immune response for survival and reproduction [23,24]. The inflammatory cell composition changes dynamically with the stage of schistosomal granuloma, regulated by cytokines, chemokines, and growth factors in complex networks. Understanding Th1/Th2 immune trends at different infection stages is crucial for studying schistosomiasis immune regulation [11,25]. During chronic schistosomiasis, egg deposition in the liver induces AAMφ to produce anti-inflammatory cytokines, creating an environment conducive to parasite survival [26,27]. Downregulating Th1 inflammatory responses can protect host tissues from *S*. *mansoni*-induced injury [28–32].

TGF-β is a key regulator of cell-cell communication with ECM. Blocking TGF-β signaling can reduce ECM synthesis in the liver. While pirfenidone, the first FDA-approved drug for idiopathic pulmonary fibrosis, blocks TGF-β expression in malignant glioma cells [33], comprehensive blockade of TGF-β might cause more severe inflammation or promote tumor formation [34,35]. Instead, short-interfering RNA (siRNA)-mediated knockdown of TGFβRI expression in HSCs could alleviate liver fibrosis induced by schistosomiasis [36]. siRNA-based therapies like Patisiran, approved by the FDA in 2018 for hereditary transthyretin-mediated amyloidosis, demonstrate the potential of RNAi therapeutics [37–39]. However, naked siRNA is prone to nuclease degradation, high renal excretion, and non-target cell uptake, leading to poor gene silencing efficiency.

Lipid nanoparticles (LNPs) are ideal carriers for nucleic acids. They contain ionizable cationic lipids, which are positive charges at low pH and neutral at physiological pH, protecting siRNA during systemic circulation [40,41] and facilitating endosomal escape upon entering the endosome [42]. LNPs with diameters smaller than 150 nm can pass through liver sinusoid fenestrations to reach the space of Disse, where HSCs reside [43–45]. However, when the particle size decreases to less than 50 nm, LNPs often experience rapid uptake by hepatocytes [46]. In this study, siRNA targeting TGFβRI (siTGFβRI) was encapsulated in LNPs to treat mice with schistosomiasis-induced liver fibrosis. The LNP-siRNA particle size was controlled between 50–100 nm for liver-targeted delivery to minimize potential side effects in other tissues. We evaluated the therapeutic potential of LNP-siTGFβRI in suppressing HSC activation *in vitro* and *in vivo*, assessing its antifibrotic effect and safety in this study.

## 2 Materials and methods

### 2.1. Ethics statement

Experiments were carried out under humane conditions with approval (license number: LAC- 2021–0516) from the Institutional Animal Care and Use Committee (IACUC) of Taipei Medical University, and conducted in accordance with the National Institutes of Health (NIH) Guide for the Care and Use of Laboratory Animals [47].

### 2.2. Chemicals and regents

The siRNA targeting human TGFβRI (sense 5’-CCA UCG AGU GCC AAA UGA AdTdT and antisense 5’-UUC AUU UGG CAC UCG AUG GdTdT) was synthesized and purified by Eurogentec (Seraing, Belgium). A non-specific scrambled (Scr) siRNA (sense 5’-AGC AUA UCA AGA CGU ACG CdTdT and antisense 5’-GCG UAC GUC UUG AUA UGC UdTdT) was used as a control. Firefly luciferase (Cat. no. L-7202) was purchased from Trilink Biotechnologies (San Diego, CA, USA). The LX-2 human hepatic stellate cell line was purchased from EMD Millipore (San Diego, CA, USA). 1,2-Distearoyl-sn-glycero-3-phosphocholine (DSPC) was purchased from Avanti Polar Lipids, Inc (Alabaster, AL, USA). Heptadecan-9-yl 8-((2-hydroxyethyl)(6-oxO-6-(undecyloxy) hexyl) amino) octanoate (SM-102), 1,2-dimyristoylrac-glycero-3-methoxypolyethylene glycol-2000 (PEG2000-DMG), and cholesterol were obtained from Sinopeg Biotech Co., Ltd. (Xiamen, Fujian, China).

### 2.3. Preparation of lipid nanoparticles

The LNPs were prepared using the NanoAssemblr Ignite (Precision Nanosystems, Vancouver, Canada). Briefly, the lipid components (SM-102, DSPC, cholesterol, and PEG2000-DMG) were fully dissolved in ethanol at a molar ratio of 50: 10:38.5:1.5 to a total lipid concentration of 25 mM total lipid. The siRNA was dissolved in 25 mM sodium acetate buffer (pH 4). The two solutions were mixed using a microfluidic chip at a total flow rate of 12 mL/min and a flow rate ratio of 3:1 (aqueous: organic phase, v/v). Nucleic acids were encapsulated at a fixed nitrogen to phosphate molar ratio of 3. The formulations were subsequently dialyzed twice against 2 L of phosphate-buffered saline (PBS, pH 7.4) using 10 kD Slide-A-Lyzer cassettes (Thermo Fisher Scientific Inc., Rockford, IL, USA) and concentrated with 10 kD Amicon Ultra-15 centrifugal filters (Millipore). The supernatants were filtered through 0.22-μm sterile polyethersulfone membranes (Pall Corp., Port Washington, NY, USA).

### 2.4. Physicochemical characterizations of lipid nanoparticles

The particle size (Z-average), polydispersity index (PDI), and zeta potential of the LNPs were measured using a Zetasizer Pro (Malvern Instruments, Worcestershire, UK). The transmission electron microscopy (TEM) images of LNPs were taken using Hitachi TEM HT-7700 (Hitachi, Tokyo, Japan). The encapsulation efficiency (EE%) of nucleic acid was measured using Quant-iT RiboGreen RNA Assay Kit (Invitrogen, Carlsbad, CA, USA). Briefly, 100 μL of diluted RiboGreen reagent was added to 100 μL of diluted LNP-siRNA in the presence or absence of 1% (w/v) Triton X-100 and incubated at room temperature for 5 min. The fluorescence intensity was measured using an M200 Pro microplate reader (Tecan, Männedorf, Switzerland) at excitation and emission wavelengths of 480 and 520 nm, respectively. EE% was calculated as follows: EE% = ((total siRNA-unencapsulated siRNA)/(total siRNA)) × 100%.

### 2.5. In vitro anti-fibrotic study

LX-2 cells (2×10^5^ cells/well) were seeded in a six-well plate containing 2 mL of DMEM (Invitrogen, Carlsbad, CA, USA) supplemented with 2% fetal bovine serum (FBS) and 1% penicillin/streptomycin (complete medium) and grown for 24 h at 37°C with 5% CO_2_. On the second day, the culture medium was removed from each well and treated with either complete medium alone, or complete medium plus 2 ng/mL of TGF-β (Sigma-Aldrich, MO, USA), or complete medium plus 2 ng/mL of TGF-β with either 100 ng/mL of LNP-Scr or LNP-siTGFβRI in a concentration ranging from 1–100 ng/mL for 48 h incubation (RT-PCR assay) or 72 h incubation (western blotting).

### 2.6. Cell uptake study

To detect the cellular uptake of LNP-siRNA, the siRNA was fluorescently labeled with a cyanine (Cy3) at the 5’ end of antisense strand. LX-2 cells were seeded in six-well plates (3×10^5^ cells/well) and cultured for 24 h at 37°C with 5% CO_2_. On the second day, cells were treated with 100 nM LNP-Cy3-siRNA and incubated for 2 h. After washing twice with PBS, 300 nM FITC-labeled LysoTracker (Invitrogen) was added to LX-2 cells for 2 h. The cells were then washed with PBS, fixed in 4% paraformaldehyde for 15 min, and the nuclei were stained with DAPI for 15 min. The fluorescence signals of Cy3, FITC, and DAPI were visualized using an EVOS M5000 microscope imaging system (Thermo Scientific) at 40×. To investigate the endocytosis mechanism in LX-2 cells, the cells were preincubated with endocytosis inhibitors (5 μM chloropromazine [CPZ], 50 μM dynasore, 25 μM ethylisopropyl amiloride [EIPA], or 1 mM methyl-β-cyclodextrin [MβCD]) for 30 min before transfection with LNP-Cy3-siRNA. For the visualization of fluorescence signals, imaging analysis of the samples was performed as described above. The inhibitors and the endocytic pathway they inhibited were shown in the S1 Table, respectively.

### 2.7. Biodistribution and ex vivo imaging

Female BALB/c mice (aged 6–8 weeks) were purchased from the National Laboratory Animal Center in Taiwan. To track the localization of LNP complexes *in vivo*, *Fluc* mRNA was used as a reporter gene for biodistribution studies. The LNP-Fluc mRNA (mRNA = 5 μg) was intravenously injected into BALB/c mice *via* the tail vein. After 6 h, the mice were intraperitoneally injected with 5 mg/mouse d-luciferin potassium salt (Caliper, Hopkinton, MA, USA). Bioluminescence signals were detected using an IVIS imaging system (PerkinElmer, MA, USA) following the injection of d-luciferin for 7 min with a constant image acquisition time of 1 min. For *ex vivo* imaging, animals were sacrificed and dissected at 6 h. Luminescence intensities in the heart, liver, spleen, lungs, and kidneys were measured using an IVIS imaging system.

### 2.8. Schistosomiasis-induced hepatic fibrosis mouse model and efficacy study

The schistosomiasis-induced liver fibrosis mouse model was developed by infecting eight-week-old female BALB/c mice percutaneously with 100–120 *S*. *mansoni* cercariae *via* the tail. The infected mice at 6 weeks post-infection were intravenously injected with PBS, LNP-Scr (0.1 mg/kg), and LNP-siTGFβRI with an siRNA dose of 0.1 and 1 mg/kg twice a week for 4 weeks. There were five mice in each group. The mice were euthanized 10 weeks post-infection, and blood and liver tissues were collected 48 h after the last administration. Blood samples were centrifuged at 3500 rpm for 15 minutes. The resulting serum supernatants were used for liver function (AST and ALT) analysis. The analysis was performed by the Laboratory Animal Center at Taipei Medical University using the IDEXX Catalyst One analyzer to measure the levels of AST (Aspartate aminotransferase, Catalog No. 98-11069-01, USA) and ALT (Alanine aminotransferase, Catalog No. 98-11067-01, USA) in the mice. Paraffin sections of liver tissue were used for hematoxylin and eosin (H&E) and Masson’s trichrome staining.

### 2.9. Real-time PCR analysis

LX-2 cells were harvested after treatment with LNP-siRNAs for 48 h *in vitro*. Livers were dissected 48 h after the last administration. The total RNA in cells and livers was extracted using Trizol reagent (Invitrogen) according to the manufacturer’s protocol. The quantity and quality of RNA were evaluated using a NanoDrop One (Thermo Fisher Scientific). Reverse transcription was performed using the High-Capacity cDNA Reverse Transcription Kit (Applied Biosystems, Foster City, CA, USA). Relative RNA expression was measured using Power SYBR Green PCR Master Mix (Applied Biosystems). The PCR thermocycling parameters were 95°C for 30 s, followed by 40 cycles of 95°C for 5 s and 60°C for 30 s, carried out using the QuantStudio 5 Real-Time PCR System (Applied Biosystems). The primer sequences used to amplify the desired cDNA were as follows: αSMA forward and reverse primers: 5′-AGGCACCCCTGAACCCCAA-3′ and 5′-CAGCACCGCCTGGATAGCC-3′; COL1A1 forward and reverse primers: 5′-GTCGAGGGCCAAGACGAAG-3′ and 5′-CAGATCACGTCATCGCACAAC-3′; TGFβRI forward and reverse primers: 5′-GACAACGTCAGGTTCTGGCTCA-3′ and 5′-CCGCCACTTTCCTCTCCAAACT-3′; and GAPDH forward and reverse primers: 5′-ATGGGGAAGGTGAAGGTCG-3′ and 5′-GGGGTCATTGATGGCAACAATA-3′. The mRNA expression of the target gene was normalized to that of *GAPDH* mRNA. Relative quantification was calculated using the double-delta method (2^−∆∆Ct^). All reactions were performed in triplicates.

### 2.10 Western blotting analysis

Western blotting was performed on the total protein extracts from LX-2 cells after treatment with LNP-siRNA for 72 h. Harvested cells were washed three times with ice-cold PBS and lysed in RIPA buffer (Thermo Fisher Scientific). Cell suspensions were centrifuged at 10,000 x g for 20 min at 4°C. The total protein concentration in the supernatant was determined using a bicinchoninic acid protein assay kit (Pierce, Rockford, IL, USA). Samples (20 μg protein) were separated using 12% SDS-PAGE gels and subsequently electrotransferred onto polyvinylidene difluoride membranes (Millipore). The membranes were blocked with 5% fat-free milk in TBST buffer at 4°C overnight. After blocking, the membranes were incubated with anti-αSMA (cat. no. PA5-87638; Invitrogen; 1:1000 dilution), anti-Collagen 1 (cat. no. PA5-95137; Invitrogen; 1:1000 dilution) and anti-TGFβRI (cat. no. PA598734; Invitrogen; 1:1000 dilution) and anti-GAPDH (cat. no. PA1-987; Invitrogen; 1:4000 dilution) at room temperature for 1 h with gentle rocking. After washing three times with TBST, the membranes were incubated with horseradish peroxidase-conjugated goat anti-rabbit secondary antibody (cat. no. BS-0295G-HRP; Bioss antibodies; 1:5000 dilution) for 1 h at room temperature. The antigen complexes were visualized using a chemiluminescent HRP detection reagent (Millipore). All western blotting experiments were performed at least three times. The blots were imaged using an Amersham Imager 600 system (GE HealthcareNJ, USA). The relative protein abundance in each sample was normalized to that of GAPDH.

### 2.11. Serum cytokines quantification

Serum cytokine levels in the terminal blood were analyzed using Quantibody Mouse Cytokine Array Q1 Kit (RayBiotech Inc., Norcross, GA, USA), which detects 20 mouse cytokines and chemokines. The samples were assayed according to the manufacturer’s instructions. The glass slides were analyzed using a laser scanner equipped with Cy3 wavelength. The fluorescence intensities were analyzed and the final cytokine concentrations (in the unit of pg/mL) were interpolated from the calibration curves.

### 2.12. Immune profiling

Whole blood was collected from BALB/c mice after 4 weeks of treatment. Cells were stained with fluorescence-conjugated antibodies for specific surface markers (CD4, CD8, CD11c, CD14, CD16, CD19, CD25, CD56, CD62L, and MHC II) and the results were analyzed using an Attune NxT Flow Cytometer (Thermo Fisher Scientific) according to the manufacturer’s instructions. The results are presented as the percentage of positive cells from the antibody of interest to the isotype control antibody.

### 2.13. Evaluation of the toxicity of LNP-siRNA in vivo

To evaluate the *in vivo* toxicity of LNP-siTGFβRI, the animals were intravenously injected with LNP-siTGFβRI (siRNA = 1 mg/kg) twice a week for four weeks, and PBS was used as a placebo. Major organs including the brain, heart, lungs, and kidneys were collected 48 h after the last administration to prepare histopathological sections. Harvested organs were fixed in 10% formalin and sectioned in paraffin for H&E staining.

### 2.14. Statistical analysis

Data were expressed as mean ± standard deviation. One-way or two-way analysis of variance (ANOVA) followed by appropriate post-hoc tests were used to determine the differences between groups. Significance for all statistical tests is shown as p<0.05 (*), p<0.01 (**), p<0.001 (***), and p<0.0001 (****) compared with the control, and p<0.05 (#), p<0.01 (##), p<0.001 (###), and p<0.0001 (####) compared with the PBS-treated control.

## 3. Results

### 3.1. Preparation and characterization of LNP-siTGFβRI

**Fig 1:**
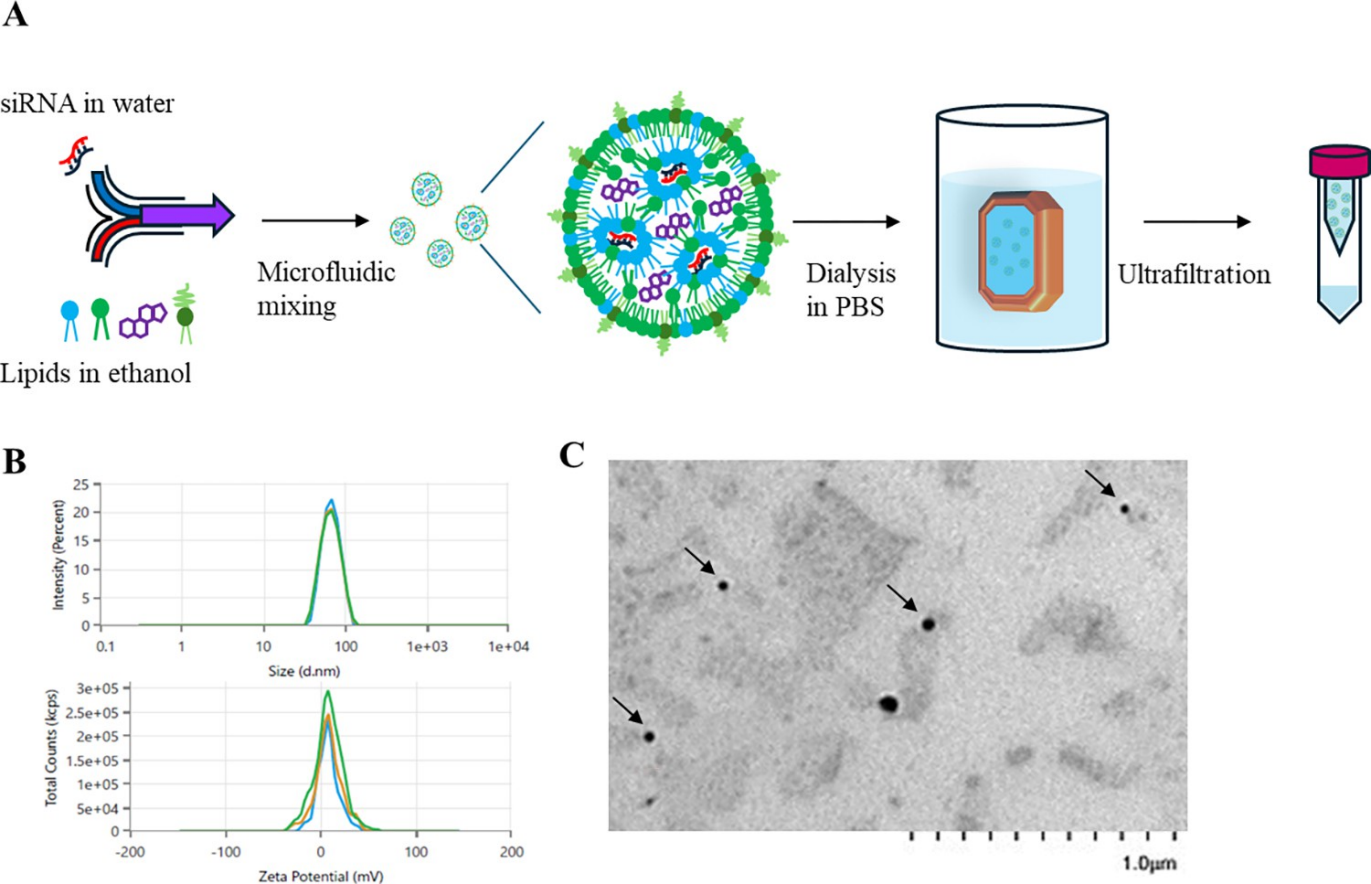
Characterization of LNP-siTGFβRI. (A) Schematic of LNP production by mixing lipid mixtures and siRNA aqueous solution via a microfluidic system. (B) Particle size distribution analysis and zeta potential intensity plots of LNP-siTGFβRI. (C) Transmission electron microscopy (TEM) image of LNP-siTGFβRI (indicated by arrows). LNP, lipid nanoparticle.

Ionizable lipid nanoparticles were produced by combining four lipid components (SM-102, DSPC, cholesterol, and PEG2000-DMG) in ethanol with siRNA in a 25 mM sodium acetate buffer (pH 4) using a microfluidic-based procedure. The LNPs were then dialyzed in PBS (pH 7.4) to remove ethanol and concentrated by ultrafiltration (Fig 1A). Dynamic light scattering measurements indicated an average particle size of 67.66 ± 0.57 nm with a polydispersity index (PDI) of 0.064 ± 0.024. The average surface zeta potential was approximately 8.84 ± 1.65 mV (Fig 1B). TEM analysis confirmed the spherical shape of the LNP-siTGFβRI cells (Fig 1C). The encapsulation efficiency was determined to be 93.92%. The biophysical attributes of the siRNA liposomal formulation used in all subsequent experiments are summarized in Table 1.

**Table 1:** Physicochemical parameters of LNP-RNAs used in the study.

LNP-RNA	Size (nm)	PDI	Zeta Potential (mV)	Encapsulation efficiency (%)
LNP-siTGFβRI	67.66 ± 0.57	0.064 ± 0.024	8.84 ± 1.65	93.92
LNP-Scr	76.39 ± 1.46	0.035 ± 0.011	0.08 ± 0.27	93.71
LNP-Cy3-siRNA	79.22 ± 0.46	0.089 ± 0.026	10.44 ± 0.66	95.00
LNP-Fluc mRNA	79.04 ± 3.77	0.057 ± 0.030	10.67 ± 2.54	96.06

LNP, lipid nanoparticle; PDI, polydispersity index.

### 3.2. In vitro activity of LNP-siTGFβRI

**Fig 2:**
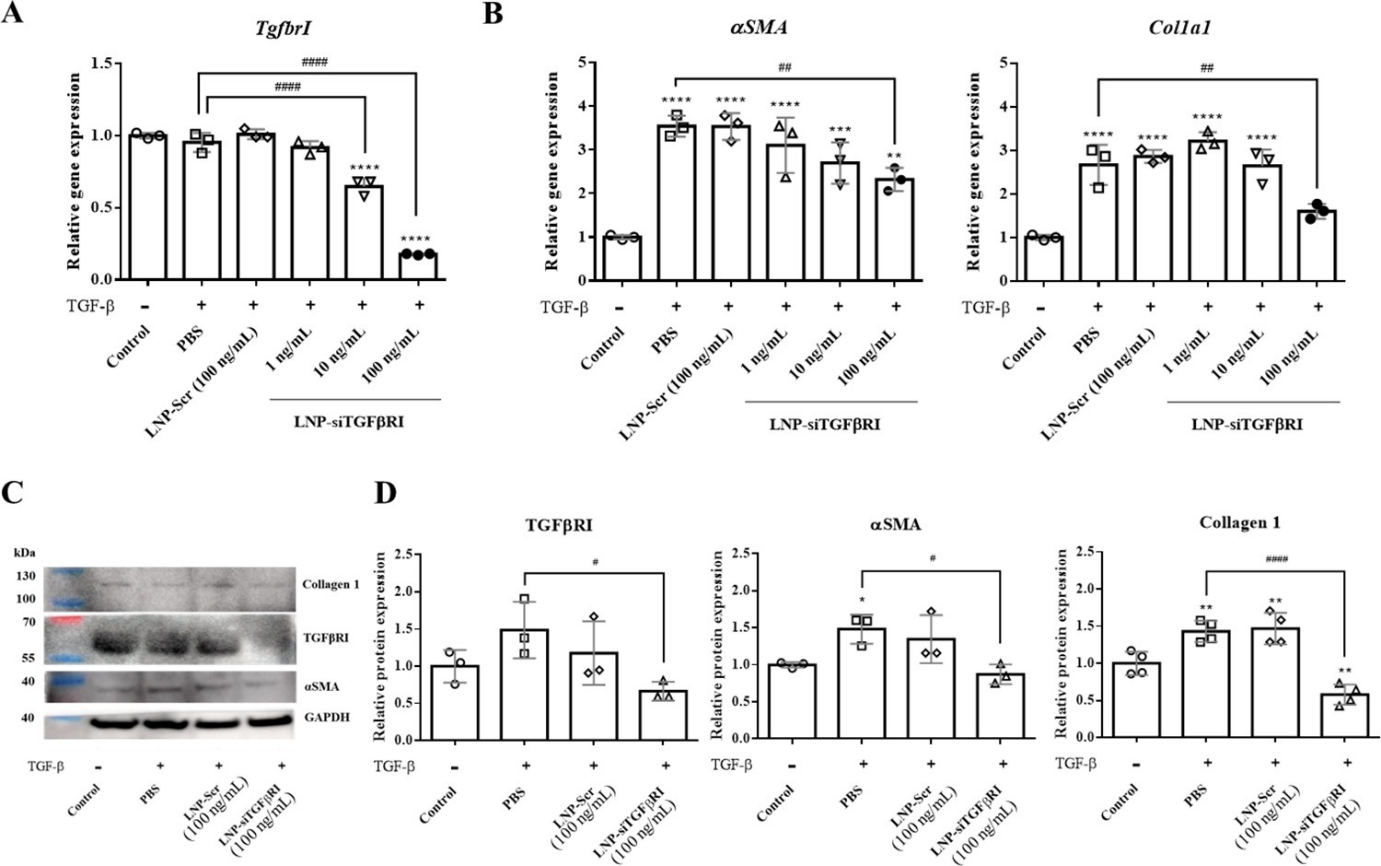
Evaluation of *in vitro* activity of LNP-siRNA on LX-2 cells stimulated by 2 ng/mL of TGF-β. (A) RT-PCR analysis of *TgfbrI* mRNA expression. (B) RT-PCR analysis of *αSMA and Col1a1* mRNA expression. (C) Western blotting (WB) analysis. (D) protein expression levels of TGFβRI, αSMA, and collagen 1 in the LX-2 cells stimulated by 2 ng/mL of TGF-β and treated with either 100 ng/mL of LNP-scrambled RNA (Scr) or LNP-siTGFβRI for 48 hours (RT-PCR) or 72 hours (WB). * p<0.05, ** p<0.01, *** p<0.001, **** p<0.0001 compared with control. # p<0.05, ## p<0.01, #### p<0.0001 indicate comparisons between two indicated groups.

To confirm whether LNP-siTGFβRI inhibited TGFβRI expression, LX-2 cells (stimulated with 2 ng/mL of TGF-β) were transfected with 1, 10 and 100 ng/mL of TGFβRI siRNA or scrambled RNA. Real-time PCR analysis at 48 hours post-transfection showed that *TgfbrI* mRNA expression levels in LNP-siTGFβRI treated groups was significantly lower at 10 and 100 ng/mL of TGFβRI siRNA compared to the PBS-treated control (Fig 2A, p<0.0001 for both). However, the effects of 1 ng/mL of TGFβRI siRNA and 100 ng/mL of scrambled RNA were not significantly different from the PBS-treated control (Fig 2A). To test whether the knockdown of TGFβRI expression would inhibit HSC activation, we measured the expression levels of the fibrotic markers *αSMA* and *Col1a1*. TGF-β treatment resulted in significant increases in the mRNA expression of *αSMA* (3.5-fold) and *Col1a1* (2.7-fold), which were blocked by TGFβRI siRNA in a concentration-dependent manner (Fig 2B). LNP-siTGFβRI significantly reduced the mRNA expression levels of *αSMA* and *Col1a1* at 100 ng/mL of TGFβRI siRNA (*αSMA*: p<0.01, *Col1a1*: p<0.01) compared to the PBS-treated control, but the effects of 1 and 10 ng/mL of TGFβRI siRNA and 100 ng/mL of scrambled RNA were not significant (Fig 2B). These results demonstrated that 100 ng/mL of LNP-siTGFβRI inhibited *TgfbrI* mRNA expression and suppressed TGF-β/SMAD signaling, reducing *αSMA* and *Col1a1* expression levels in TGF-β-stimulated LX-2 cells. The TGFβRI, αSMA and collagen 1 protein expression levels in LX-2 cells were confirmed by western blotting after treatment with either 100 ng/mL of LNP-Scr or LNP-siTGFβRI in the presence of 2 ng/mL of TGF-β for 72 hours (Fig 2C). TGFβRI siRNA significantly reduced the expression of TGFβRI, αSMA, and collagen 1 in LX-2 cells (TGFβRI: p<0.05, αSMA: p<0.05, collagen 1: p<0.0001) compared to the PBS-treated control, whereas the effect of 100 ng/mL of LNP-Scr was not significant (Fig 2D).

### 3.3. Cellular uptake property of LNP-siRNA

**Fig 3:**
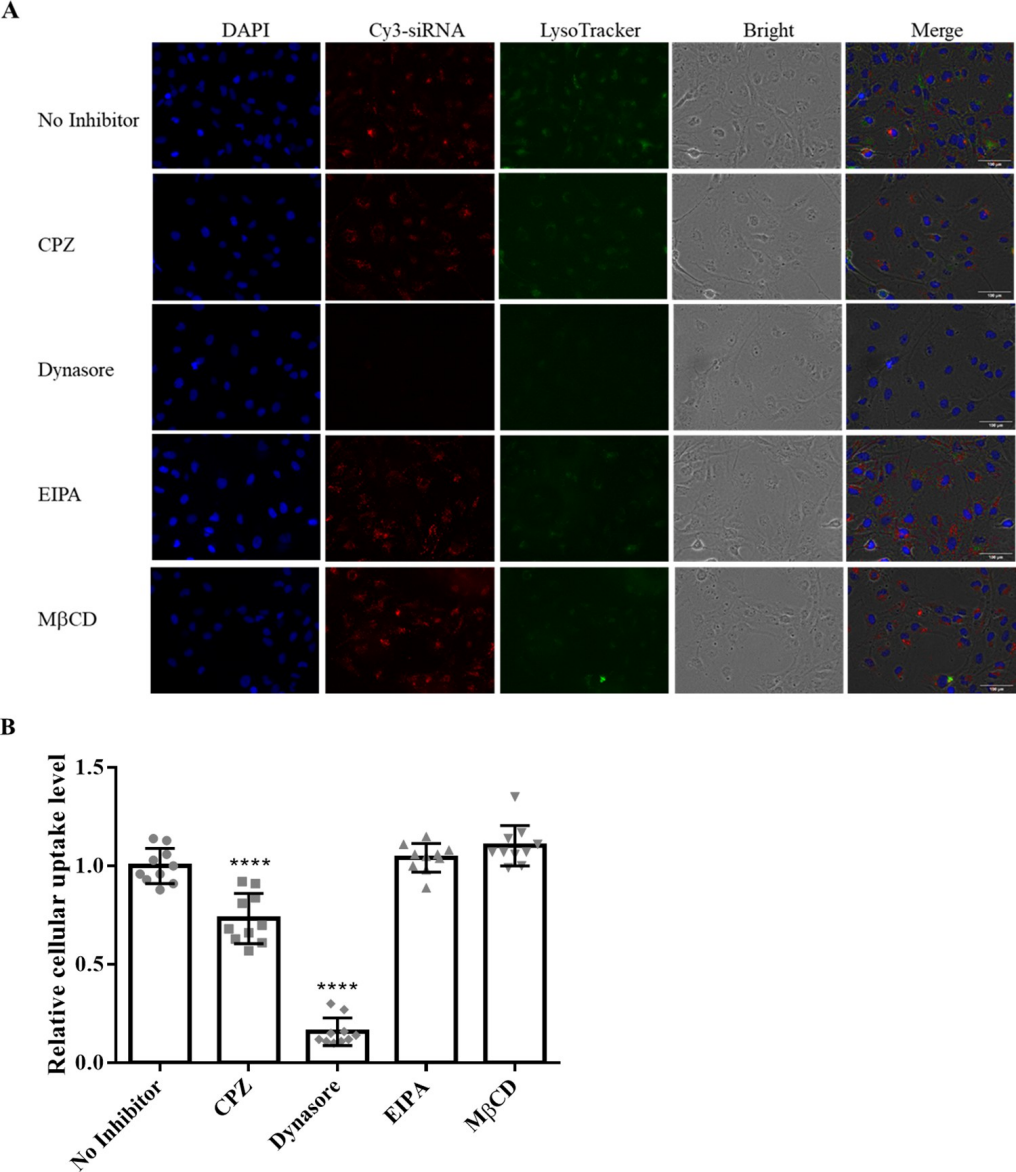
Cellular uptake properties of LNP-siRNA by LX-2 cells. (A) Fluorescence microscopy images of LX-2 cells incubated with LNP-Cy3 labeled siRNA (red), LysoTracker (green), and DAPI (blue) in presence of endocytic inhibitors CPZ, Dynasore, EIPA, and MβCD. (B) Analysis of the endocytosis mechanisms of LNP-siRNA by LX-2 cells in presence of various endocytic inhibitors. **** p<0.0001 compared with untreated control. LNP, lipid nanoparticle.

LX-2 cells were incubated with LNP-Cy3-siRNA for 2 hours. Cell lysosomes and nuclei were stained with LysoTracker Green and DAPI (blue), respectively, and cellular uptake was observed using fluorescence microscopy. The images showed that Cy3-siRNA (red) was widely distributed in the cytoplasm, with locations not completely overlapping with the lysosomes (green). This suggests that LNP-Cy3-siRNA was effectively taken up by LX-2 cells and that Cy3-siRNA efficiently escaped from the lysosomes (Fig 3A). To further investigate the internalization mechanism, LX-2 cells were treated with various endocytic pathway inhibitors for 30 minutes before transfection with LNP-Cy3-siRNA. CPZ and Dynasore inhibit clathrin-mediated and dynamin-dependent endocytosis, respectively, while EIPA and MβCD inhibit macropinocytosis and caveolin-mediated endocytosis, respectively. As illustrated in Fig 3A and 3B, the uptake of LNP-Cy3-siRNA in LX-2 cells was significantly inhibited by CPZ and Dynasore, but not by EIPA and MβCD (p<0.0001). This indicates that the uptake of LNP-siRNA by LX-2 cells is likely mediated by clathrin- and dynamin-dependent endocytosis.

### 3.4. Biodistribution of LNP particle size between 50 and 100 nm

**Fig 4:**
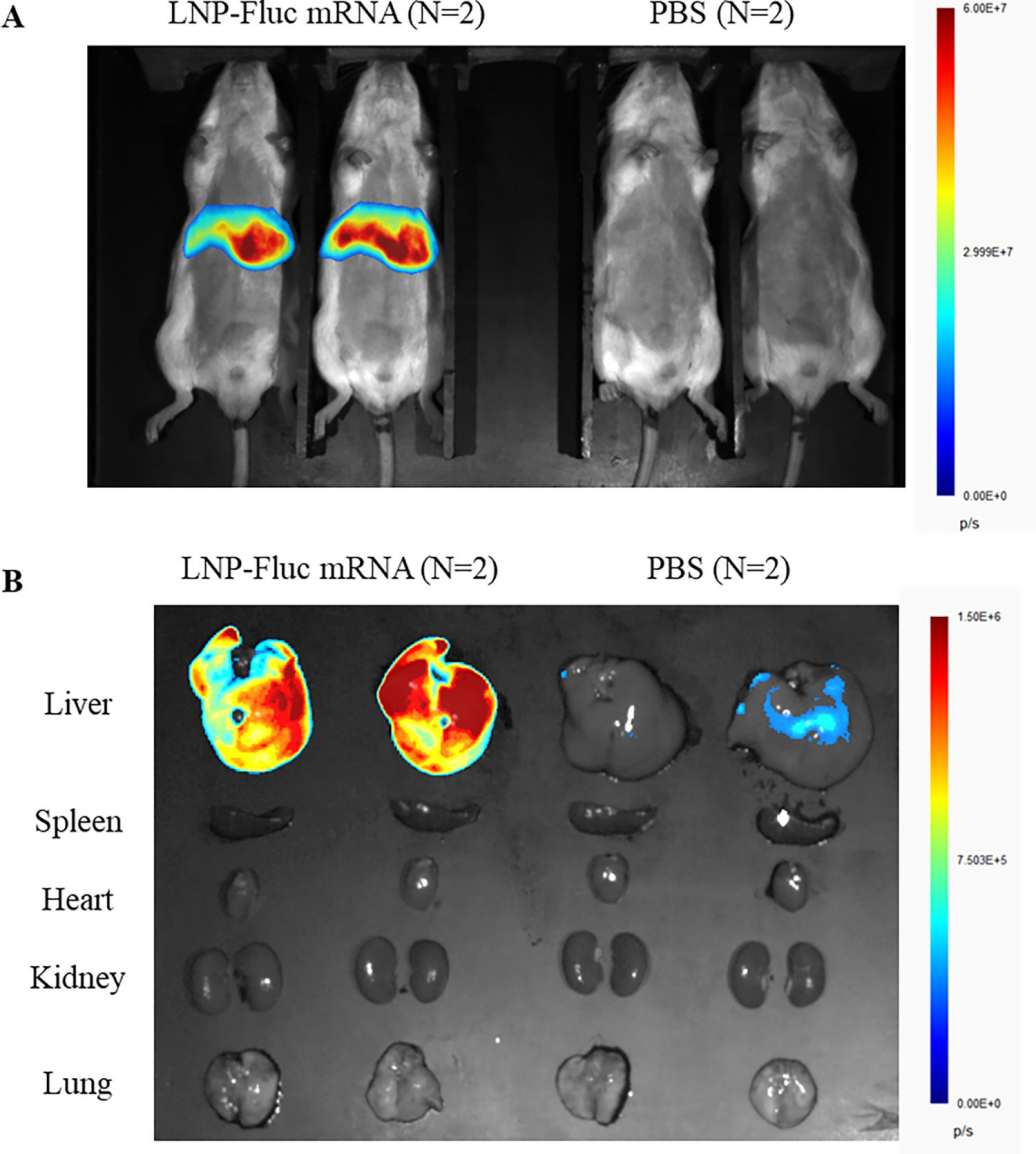
*In vivo* specific delivery and biodistribution of LNP-Fluc mRNA. (A) Bioluminescent image of BALB/c mice administrated with LNP-Fluc mRNA (particle size = 79.04 ± 3.77 nm) via intravenous injection. (B) *Ex vivo* images of major organs excised from mice 6 hours post injection. LNP, lipid nanoparticle.

An *in vivo* bioluminescence imaging study was conducted to investigate the distribution of 50–100 nm LNPs in BALB/c mice. After intravenous injection of LNP-Fluc mRNA (5 μg mRNA), bioluminescent signals in the mice were detected at 6 hours post-injection using an imaging system. The results showed that the bioluminescence intensity was primarily concentrated in the liver (Fig 4A). For a more precise analysis, major organs (liver, spleen, heart, kidney, and lungs) were harvested 6 hours post-injection for *ex vivo* imaging. As shown in Fig 4B, bioluminescence signals were detected only in the liver, clearly indicating the high liver specificity of LNPs with a size of 50–100 nm.

### 3.5. LNP-siTGFβRI ameliorates S. mansoni-induced liver fibrosis

**Fig 5:**
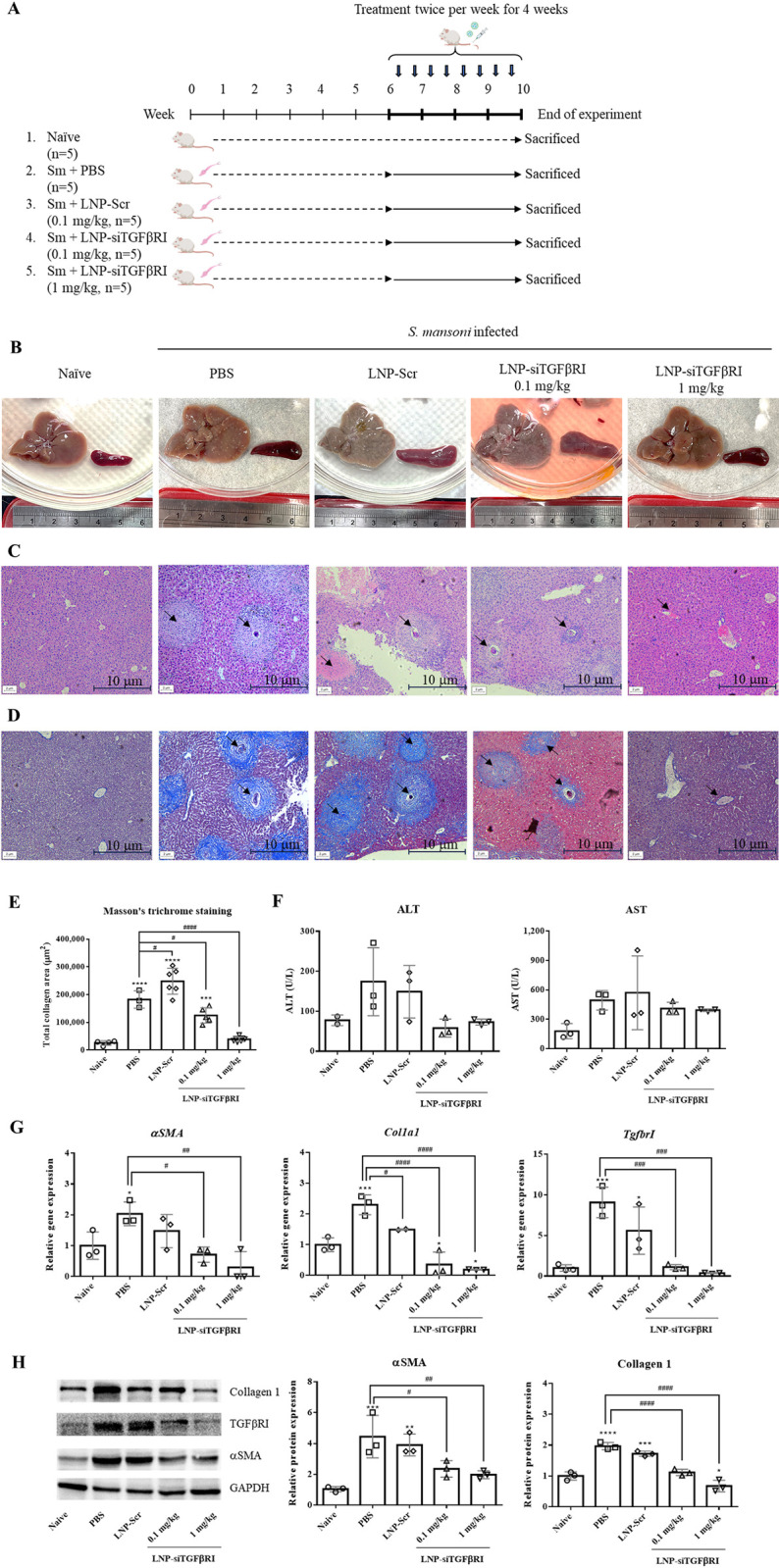
Effect of intravenous injection of LNP-siTGFβRI on *Schistosoma mansoni*-induced liver fibrosis in mice. (A) Scheme illustrating the experimental design for the *in vivo* study. (B) Macroscopic appearances of hepatic and splenic tissues from naïve and infected mice treated with PBS, LNP-Scr, and LNP-siTGFβRI at the end of the experiment after 10 weeks of infection. (C) H&E staining of liver sections, shown at 100× magnifications. (D) Masson’s trichrome staining of liver sections, shown at 100× magnifications. (E) Granuloma size measured from trichrome staining of liver sections. (F) ALT and AST levels in serum. (G) Real-time PCR analysis of mRNA levels of *αSMA*, *Col1a1*, and *TgfbrI* in liver tissues. (H) Western blotting analysis of protein levels of αSMA, collagen 1, and TGFβRI in liver tissues. Data are expressed as the mean ± standard deviation (SD). The arrows indicate the worm egg locations. * p<0.05, *** p<0.001, **** p<0.0001 compared with naïve mice. # p<0.05, ## p<0.01, ### p<0.001, #### p<0.0001 indicate comparisons between two indicated groups. LNP, lipid nanoparticle; Scr, scrambled RNA.

To investigate the therapeutic effect of LNP-siTGFβRI on schistosomiasis-induced liver fibrosis in mice, the animals were randomly divided into four groups (n = 5) and intravenously injected with PBS, 0.1 mg/kg/dose of LNP-Scr, 0.1 mg/kg/dose of LNP-siTGFβRI, or 1 mg/kg/dose of LNP-siTGFβRI twice a week from the sixth to the tenth weeks after infection. A schematic of the *S*. *mansoni*-induced liver fibrosis mouse model and its treatment is shown in Fig 5A. The numbers of infected worm burden in each group were respectively revealed in the S2 Table. The results showed that the numbers of recovered worms were also reduced under the influence of LNP-siTGFβRI. The gross pathology of the abdominal lesions was examined 10 weeks after infection. Macroscopically, granuloma nodules spread over the liver surface, and severe hepatic fibrosis was observed in the PBS-treated and LNP-Scr-treated groups. The nodular appearance on the liver gradually disappeared with increased doses of LNP-siTGFβRI (0.1 and 1 mg/kg of siRNA) for 4 weeks (Fig 5B). Histologically, eggs were observed in the livers of *S*. *mansoni*-infected mice. Marked inflammatory infiltrates surrounding the trapped eggs formed granulomas in H&E-stained sections of the PBS-treated and LNP-SC-treated groups (Fig 5C). In contrast, the livers of infected mice treated with 0.1 mg/kg of LNP-siTGFβRI had few and small granulomas, and the liver sections of mice treated with 1 mg/kg of LNP-siTGFβRI were nearly free of granulomas (Fig 5C). Consistent with the H&E staining results, Masson’s trichrome staining showed reduced collagen expression around the trapped eggs in the liver sections of the LNP-siTGFβRI-treated groups compared to the PBS- and LNP-Scr-treated groups (visualized as blue in Fig 5D). Image analysis showed that the collagen deposition area significantly decreased following treatment with LNP-siTGFβRI in a dose-dependent manner compared to the PBS-treated group (p<0.05 and p<0.0001, respectively) (Fig 5E). Additionally, mice infected with *S*. *mansoni* showed increased ALT and AST levels in the serum of the PBS- and LNP-Scr-treated groups compared to the uninfected group. The ALT and AST levels in the LNP-siTGFβRI-treated groups showed a decreasing trend, but were not statistically significant compared to the PBS-treated group (Fig 5F). The mRNA and protein levels of liver fibrosis markers *αSMA* and *Col1a1* were investigated in excised liver tissues. PCR results showed that the mRNA levels of *αSMA*, *Col1a1*, and *TgfbrI* in *S*. *mansoni*-infected mice (PBS-treated group) were increased compared to the uninfected group (p<0.05). Following LNP-siTGFβRI treatment, the mRNA levels of fibrosis markers were significantly reduced in a dose-dependent manner compared to the PBS-treated group (p<0.05 and p<0.01, respectively) (Fig 5G). The changes in *αSMA* and *TgfbrI* were not significant between the PBS-treated and LNP-Scr-treated groups. Consistent with the RT-PCR results, western blot analysis showed significantly reduced expression levels of αSMA (0.1 mg/kg: p<0.05, 1 mg/kg: p<0.01), collagen 1 (0.1 mg/kg: p<0.0001, 1 mg/kg: p<0.0001) and TGFβRI in the LNP-siTGFβRI-treated groups compared to the PBS- and LNP-Scr-treated groups (Fig 5H).

### 3.6. LNP-siTGFβRI regulates cytokine responses and cellular responses of S. mansoni-infected mice

**Fig 6:**
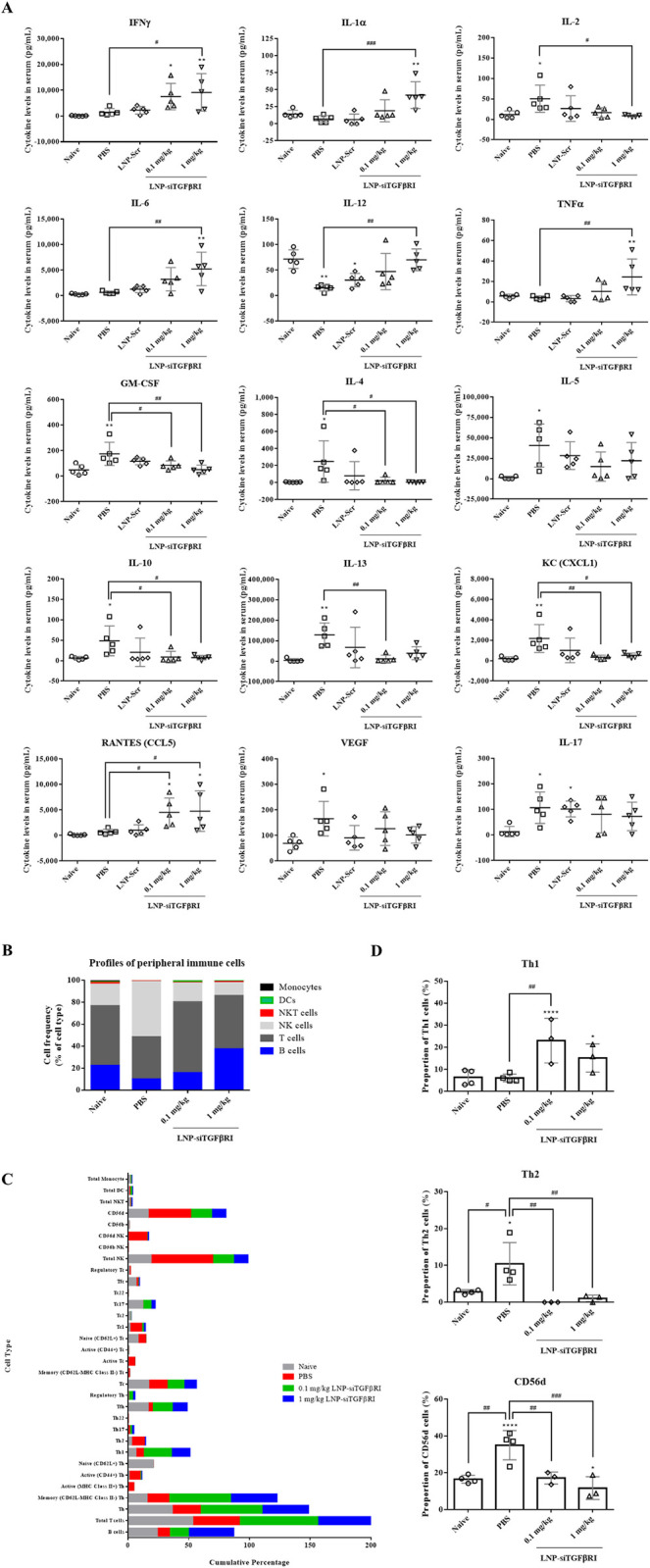
Serum cytokine concentrations and venous immune cell profiles in naïve and *S*. *mansoni*-infected mice at 10 weeks post-infection. (A) Concentrations of Th1/Th2 cytokines in the serum. (B) Stacked bar charts showing cell frequencies as a proportion of total live cells at 10 weeks post-infection. Data are presented with averages calculated from experiments. (C) Cumulative charts showing differences in cell frequencies among naïve, PBS-treated, and LNP-siTGFβRI-treated mice. (D) The frequencies of Th1, Th2 and CD56dNK cells in different groups were calculated, respectively. Data are expressed as the mean ± standard deviation (SD). * p<0.05, ** p<0.01 compared with naïve mice. # p<0.05, ## p<0.01, ### p<0.001 indicate comparisons between two indicated groups.

To further elucidate the liver-protective effects of LNP-siTGFβRI in *S*. *mansoni* infected mice, venous blood was collected 10 weeks post-infection to measure serum cytokine concentrations and analyze the profiles of immune cells in naïve and *S*. *mansoni*-infected mice. In the serum cytokine assay, *S*. *mansoni-*infected mice (PBS-treated group) showed decreased levels of IL-12 (a Th1 cytokine) and increased levels of GM-CSF, IL-4, IL-5, IL-10, IL-13, KC (CXCL1), and VEGF (Th2 cytokines) compared to naïve mice (p<0.05) (Fig 6A), indicating a Th2-biased immune response during chronic schistosome infection, despite an unexpected increase in IL-2 levels. Upon treatment with LNP-siTGFβRI, there wea an increase in Th1 cytokines, such as IFNγ, IL-1α, IL-6, IL-12, and TNFα (p<0.05), and a concurrent decrease in Th2 cytokines, such as GM-CSF, IL-4, IL-10, IL-13, and KC (p<0.05) (Fig 6A). Except for IL-2, Th1 cytokine levels were elevated in the LNP-siTGFβRI-treated groups compared to the PBS-treated group, while the LNP-Scr-treated group did not show significant changes in cytokine expression compared to the PBS-treated group.

Additionally, we identified the immune cell types responding to venous blood from control and treated mice. At 10 weeks post-infection, PBS-treated mice exhibited a significant decrease in CD19^+^B cells and total T cells, and an increase in total NK cells compared to naïve mice (Fig 6B). The distribution proportions of different cell types and their associations with the various treatments are shown in Fig 6C. Notable changes included a decrease in B cells and follicular helper T (Tfh) cell populations and an increase in Th2 and CD56d NK cell proportions from naïve to PBS-treated infected mice (S3 Table). Following LNP-siTGFβRI treatment, there was an upregulation of B and Th1 cell populations, and a downregulation of CD56d NK cells. Our data showed Th2 cells and CD56dNK were both significantly declined after LNP-siTGFβRI treatment (p<0.05) (Fig 6D). Overall, egg production induced Th2-cell and CD56d NK cell-dominated immune responses. The expression of GM-CSF, IL-4, IL-5, IL-10, IL-13, KC, and VEGF in Th2 cells, and IL-2, IL-4, IL-5, IL-10, and IL-17 in NK cells significantly increased after infection (PBS-treated group). The knockdown of TGFβRI affected HSC activation and impacted Th2-cell and CD56d NK-cell development. Consequently, LNP-siTGFβRI-treated mice exhibited a Th1-skewed response and a decreased percentage of CD56d NK cells compared to the PBS-treated group (Fig 6C and 6D).

### 3.7. In vivo toxicity of LNP-siTGFβRI

**Fig 7:**
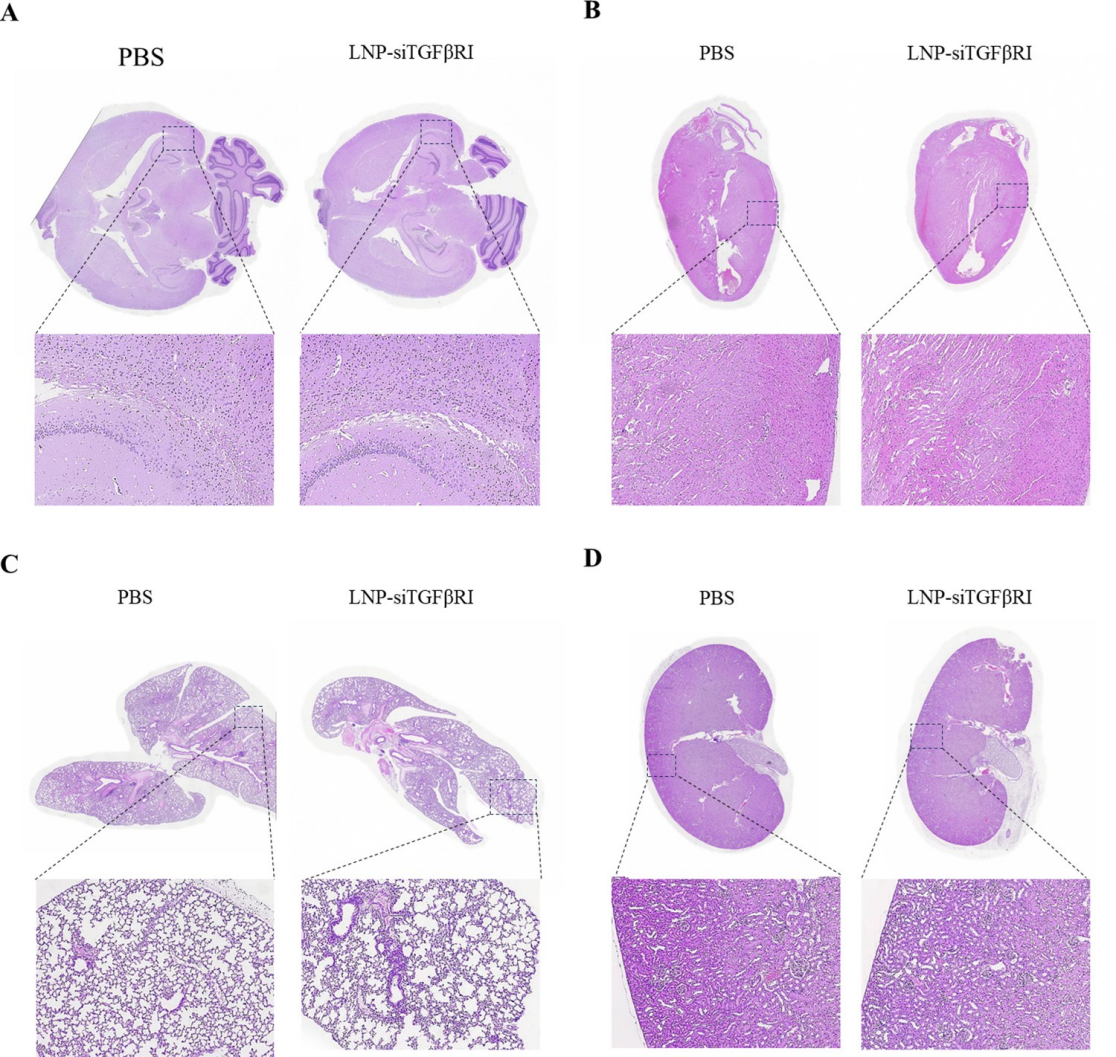
Histopathological examination (H&E) of the (A) brain, (B) heart, (C) lung, and (D) kidney collected at 48 h after the last administration of LNP-siTGFβRI (1 mg/kg/dose) injected twice a week for 4 weeks, or PBS. Images were taken with a 10× objective lens.

To further evaluate the *in vivo* toxicity of LNP-siTGFβRI, we assessed changes in tissue histology in mice. Healthy BALB/c mice were treated with PBS and LNP-siTGFβRI at a dose of 1 mg/kg, following the same schedule used in the *in vivo* efficacy study. Major organs were collected from the treated mice for histopathological examination. H&E staining revealed no obvious pathological changes in the brain, heart, lung, and kidney in both the PBS- and LNP-siTGFβRI-treated groups (Fig 7A–7D). These data demonstrated the safety of LNP-siTGFβRI in mice.

## 4 Discussion

Inflammatory cytokine TGF-β, released from hepatocytes and macrophages, promotes liver fibrosis by stimulating the activation of HSCs. TGF-β binds to the transmembrane TGFβR complex and elicits downstream signaling. There are two functionally distinct types of TGFβRs: TGFβRI and TGFβRII. TGF-β directly binds to TGFβRII. Once bound, the TGFβRII complex recruits the TGFβRI dimer and phosphorylates the serine and threonine residues in the cytosolic domain of TGFβRI, triggering the signaling cascade involving Samd2, Smad3, and Smad4. This cascade induces the transcription of targe genes such as αSMA and type I and III collagen [14,15]. However, studies have shown that the ligand-bound TGFβRII complex interacting with Activin receptor-like kinase 1 (ALK1) has opposing functions compared to the TGFβRI kinase [48–50]. Thus, TGFβRII appears to act as a gatekeeper rather than a direction-determining factor in the TGF-β signaling pathway [51]. Numerous studies have demonstrated that downregulating TGFβRI expression can help prevent fibrosis [52–54]. In our study, we showed that knockdown of TGFβRI using lipid-encapsulated siRNA against TGFβRI effectively reduced TGF-β-induced fibrotic responses in human LX-2 cells *in vitro*. Treatment with LNP-siTGFβRI in a mouse model of schistosomiasis led to significant histological improvement, with decreased collagen deposition in the granulomatous lesion regions.

LNPs are rapidly distributed throughout the systemic circulation following intravenous injection. In most mammals, the liver sinusoidal endothelium features open fenestrations with pore sizes of approximately 200 nm [55]. Numerous studies have shown that particles small than 150 nm can pass through these sinusoidal fenestrae and reach the space of Disse [49–51]. The LNPs used in the study had particle size ranging from 50 to 100 nm, a PDI of less than 0.09, indicating monodispersity and ideal dispersion, and a neutral to mildly cationic charge after RNA loading. The established mechanism for LNP internalization is through the conserved endosomal trafficking pathway [56], which includes clathrin-mediated, dynamin-dependent, macropinocytosis, and caveolin-mediated pathways [57,58]. In our cellular uptake study, we treated LX-2 cells with LNP-Cy3-siRNA and various endocytosis pathway inhibitors to investigate their endocytotic ability and mechanism of internalization. The results indicated that LNP-Cy3-siRNA successfully entered the cytosol and was inhibited by CPZ and Dynasore, suggesting that the endocytosis of LNP-siRNA into LX-2 cells was mediated by clathrin- and dynamin- dependent mechanisms. The *in vivo* biodistribution of LNP-Fluc mRNA identified the liver as the predominant target organ, with detectable bioluminescence revealing that the RNA cargo escaped from lysosomes and became functional in the cytosol of the target cells.

Treatment with LNP-siTGFβRI in *S*. *mansoni*-infected mice during the granuloma formation and chronic fibrosis phases of schistosomiasis showed significant overall improvement. After intravenous injections of LNP-siTGFβRI twice a week for a total of eight doses (0.1 and 1 mg/kg/dose), the treatment successfully knocked down TGFβRI mRNA in the liver tissues of disease mice. RT-PCR analysis indicated that mRNA levels of the fibrotic markers *aSMA* and *Col1a1* were significantly suppressed in the liver tissues of LNP-siTGFβRI-treated groups. The fibrotic region area was significantly reduced by LNP-siTGFβRI treatment, whereas LNP-Scr treatment failed to alleviate liver fibrosis. A small-molecule inhibitor of TGFβRI, Vactosertib, has been reported to highly specifically inhibit TGF-β/SMAD signaling to exert its anti-fibrotic activity without exhibiting toxicity in mice at therapeutic doses [59,60]. However, it revealed little toxicity in a preclinical study with 4-week-old rats at doses up to 120 mg/kg [61]. Histopathological examination showed no obvious pathological changes in the major organs of LNP-siTGFβRI-treated mice compared to the PBS group, indicating good *in vivo* biosafety of LNP-siTGFβRI.

We observed decreased IL-12 (a Th1 cytokine) and increased levels of GM-CSF, IL-4, IL-5, IL-10, IL-13, KC (CXCL1), and VEGF (Th2 cytokines) in the serum of PBS-treated mice compared to naïve mice. Infection with *S*. *mansoni* alters Th1 and Th2 cytokine profiles. Following parasite oviposition, the host mounts a predominantly type 2 immune response, characterized by reduced production of Th1 cytokines and increased Th2 cytokines [62,63]. However, treatment with LNP-siTGFβRI shifted this Th2-predominant cytokine profile. In the serum of LNP-siTGFβRI-treated mice, we observed higher levels of IFNγ, IL-1α, IL-6, IL-12 and TNFα (Th1 cytokines) and suppressed level of GM-CSF, IL-4, IL-10, IL-13, and KC (Th2 cytokines) compared to the untreated control group (PBS). These cytokines regulate the progression of liver fibrosis, with Th1 cytokines inhibiting HSC activation [64,65] and Th2 cytokines promoting it [66,67]. Following LNP-siTGFβRI treatment, the Th1-skewed immune polarization exhibited an anti-fibrotic effect on schistosomiasis-induced liver fibrosis. Notably, we found that IL-2, a Th1 cytokine, behaved oppositely: it increased in the untreated group (PBS) and decrease in the LNP-siTGFβRI-treated group. IL-2 promotes the differentiation of naïve T cells into Th1 cells and is crucial for the proliferation of NK cells [68]. Li *et al*. suggested that NK cells play a significant role in host defense against *S*. *japonicum* infection [69,70]. Similarly, our immune cell profiling assay showed that total NK cells increased in untreated *S*. *mansoni-*infected mice compared to naïve mice. Following LNP-siTGFβRI treatment, HSC activation was suppressed, and the NK cell population was downregulated. Our findings indicate that NK cells may also play important roles in inflammation and immune response to *S*. *mansoni* infection. Meanwhile, previous studies also found that *Schistosoma mansoni* infection induces plasmablast and plasma cell death in the bone marrow, and our study also showed similar results in the blood, which were restored B cells after LNP-siTGFβRI treatment [71].

**Fig 8:**
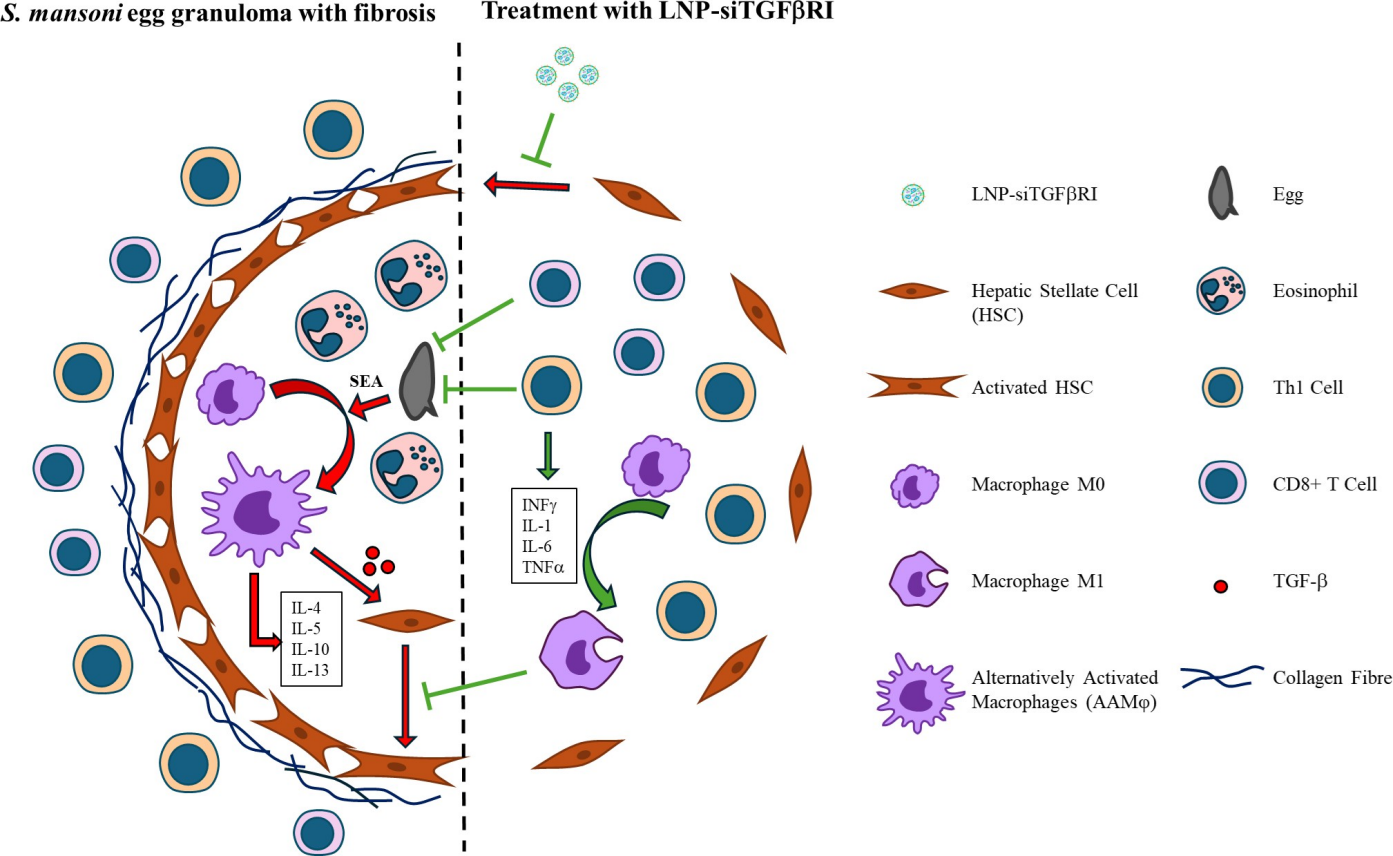
Proposed mechanism of LNP-siTGFβRI action on schistosomiasis-induced liver fibrosis. *S*. *mansoni* eggs release SEA, which promotes TGF-β production from AAMφ. TGF-β significantly activates hepatic stellate cells and increases collagen production. LNP-siTGFβRI inhibits the TGF-β/SMAD signaling pathway, reducing extracellular matrix levels. This process also influences the infiltration of CD4+ T cells and CD8+ T cells, shifting the immune response from Th2 to Th1 polarization, thereby aiding in egg elimination.

Numerous studies have reported that SEA secreted by *S*. *mansoni* eggs can polarize macrophages into AAMφ phenotypes [18,72,73]. These AAMφs overproduce TGF-β, which promotes the activation of HSCs. In this study, we demonstrated that liver fibrosis caused by *S*. *mansoni* infection could potentially be inhibited by LNP-siTGFβRI. The possible mechanisms by which LNP-siTGFβRI suppresses liver fibrosis include down-regulating the activation of HSCs, leading to a reduction in ECM levels, and modulating the granuloma microenvironment by shifting Th2 cell polarization towards Th1 cells. The increase in Th1 cytokines resulted in enhanced CD8+ T cell infiltration [74], which in turn facilitated the elimination of the eggs (Fig 8).

We acknowledge several limitations in our study. First, we did not collect single-cell suspensions from the liver and spleen. Second, we did not examine the relative mRNA expression levels of cytokines in the liver and spleen. As a result, we were unable to compare immune cell compositions and cytokine profiles between systemic circulation and various effector sites. Lastly, our efficacy study design did not include additional treatment time points, such as dosing for two weeks from the sixth to the eighth weeks post-infection as a parallel treatment group. These limitations will be improved in our subsequent studies.

## 5. Conclusion

In this study, we constructed the LNP-siTGFβRI with potent inhibition of type I TGF-β receptor expression in both LX-2 cells and *S*. *mansoni*-infected mouse liver, which led to downregulation of fibrogenic genes *aSMA* and *Col1a1*. The markedly decreased levels of extracellular matrix proteins resulted in significant relief of hepatic fibrosis induced by *S*. *mansoni*. The mechanism may involve enhancement of type 1 cellular immunity (involving NK cell activation), thereby suppressing fibrosis-related Th2 responses. Furthermore, the LNP-siTGFβRI exhibited a safe profile *in vivo*. These results indicated that LNP-siTGFβRI may be an effective and specific therapeutic strategy for treating liver fibrosis caused by *S*. *mansoni* infection.

## Supporting information

S1 Table Different inhibitors and their endocytic pathways inhibited. * indicates pathways that may mediate LNP-siRNA endocytosis.
(TIF)

S2 Table The worm recovery burden and reduction rate of each group were calculated in the study. * p < 0.05, ** p < 0.01, as compared to PBS positive control group.
(PDF)

S3 Table Statistics of immune cell frequencies of treatments and their comparison to naïve mice. Arrows in table represent the direction of cell frequency change in infected animals in comparison to naïve. * p < 0.05, ** p < 0.01, *** p < 0.001, **** p < 0.0001, ns = non-significant (p > 0.05) compared with naïve group.
(PDF)

S1 Data Raw data of the results in all figures. (ZIP)
