# Supplementary figures and images for: Development of a Lipid-encapsulated TGFβRI-siRNA Drug for Liver Fibrosis Induced by Schistosoma mansoni
Source: PLoS Negl Trop Dis. 2024 Sep 12;18(9):e0012502. doi: 10.1371/journal.pntd.0012502 (PMC11421824; doi:10.1371/journal.pntd.0012502)

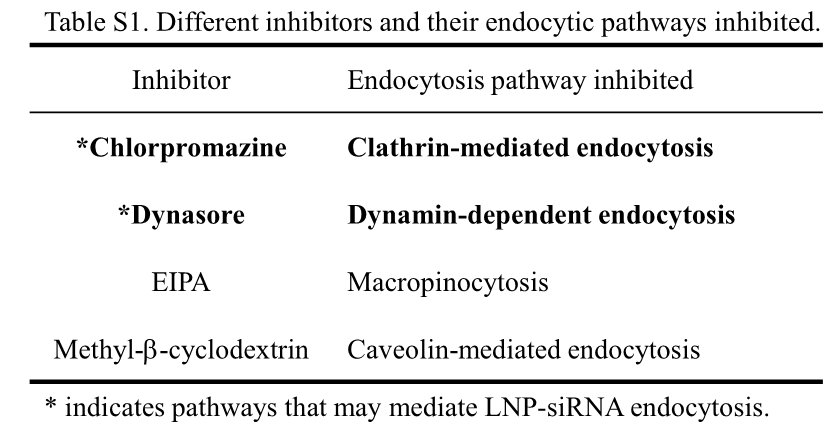

Supplement: S1 Table — * indicates pathways that may mediate LNP-siRNA endocytosis. (TIF) [file pntd.0012502.s001.tif]

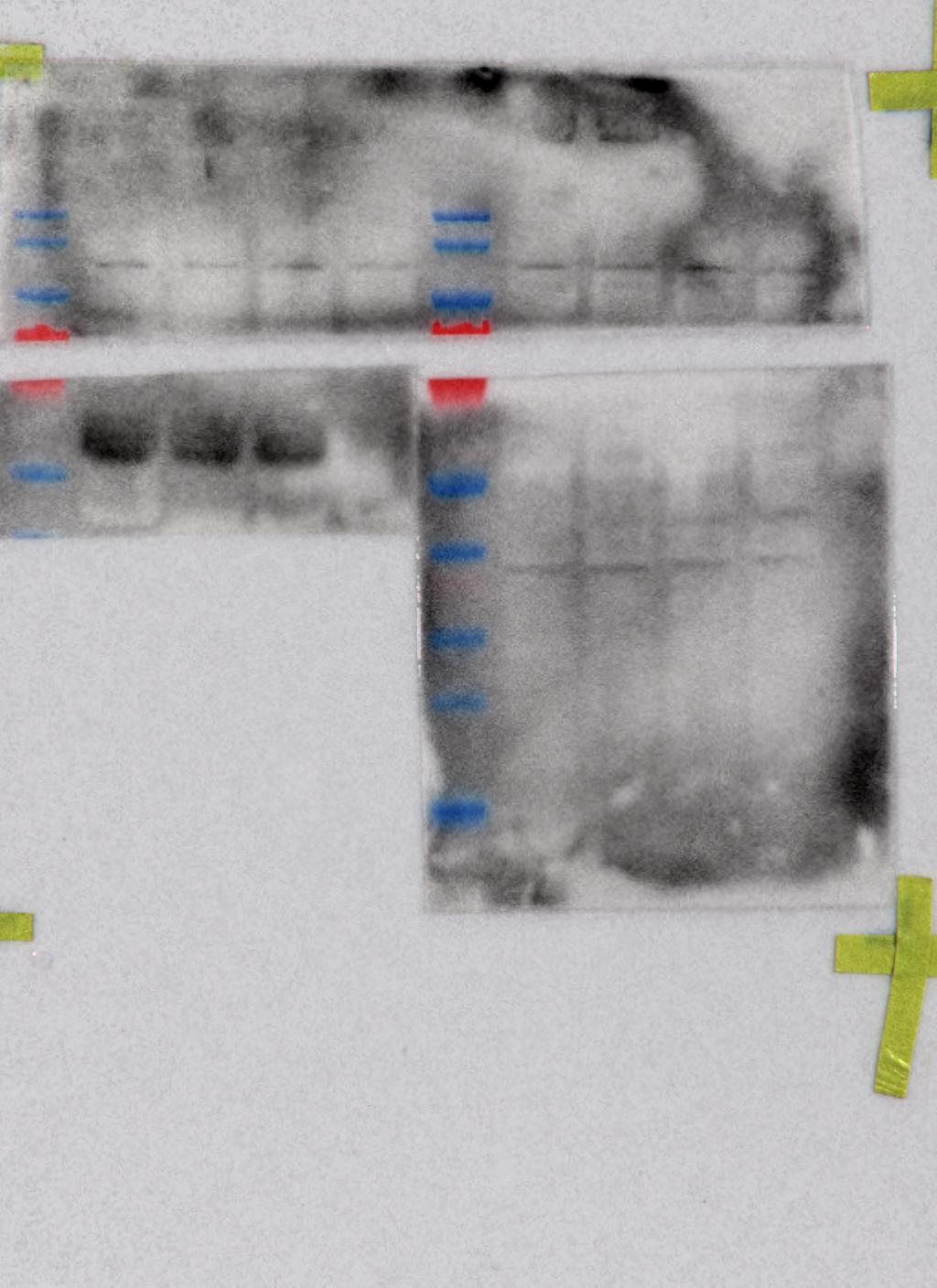

Supplement: S1 Data — (ZIP) [file pntd.0012502.s004.zip › Raw data/fig.2 WB raw data/20230329 WB Col(up-left)_TGFbRI(down-left)_aSMA(down-right).jpg]

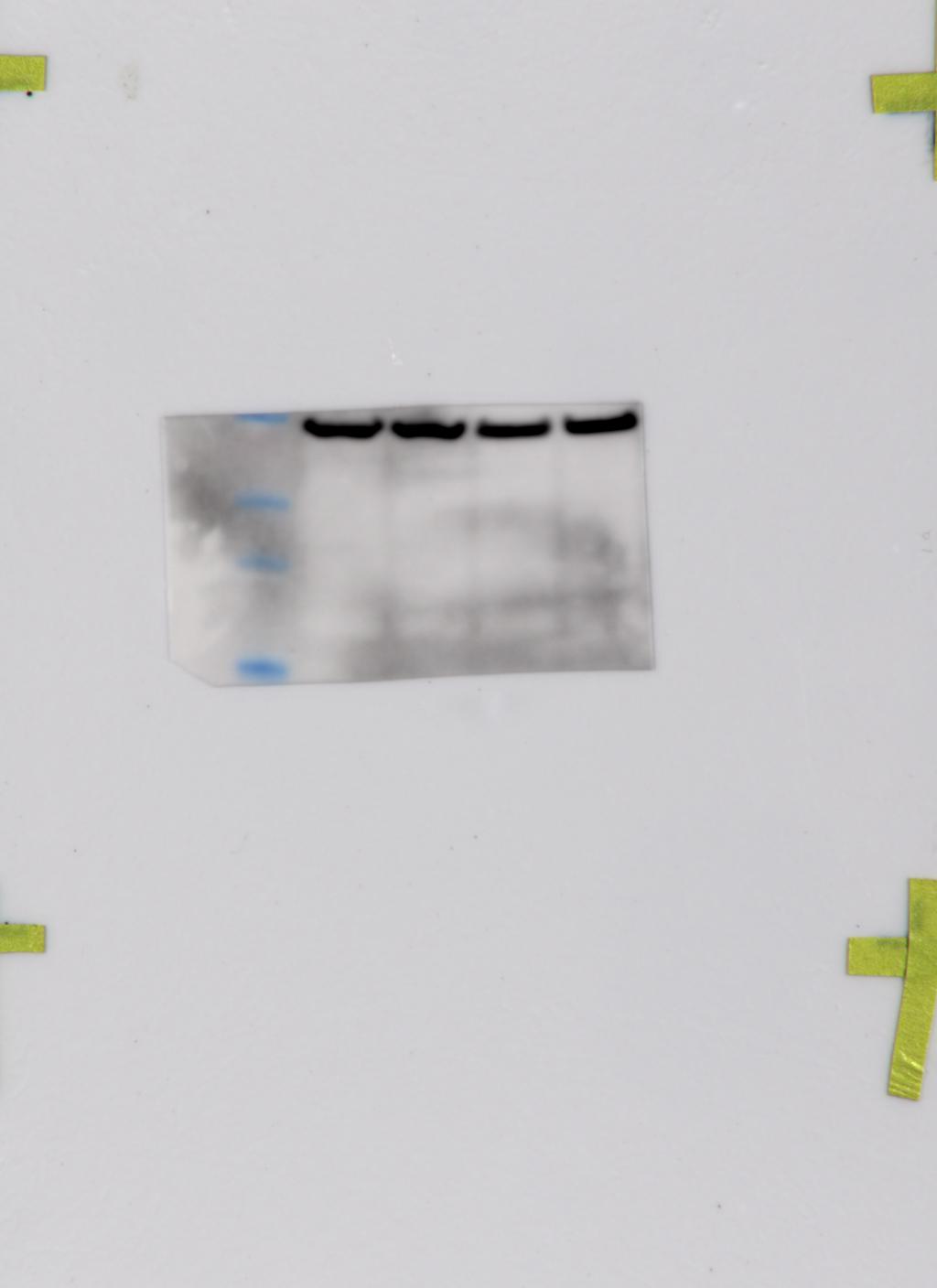

Supplement: S1 Data — (ZIP) [file pntd.0012502.s004.zip › Raw data/fig.2 WB raw data/20230329 WB GAPDH.jpg]

## Slide 1
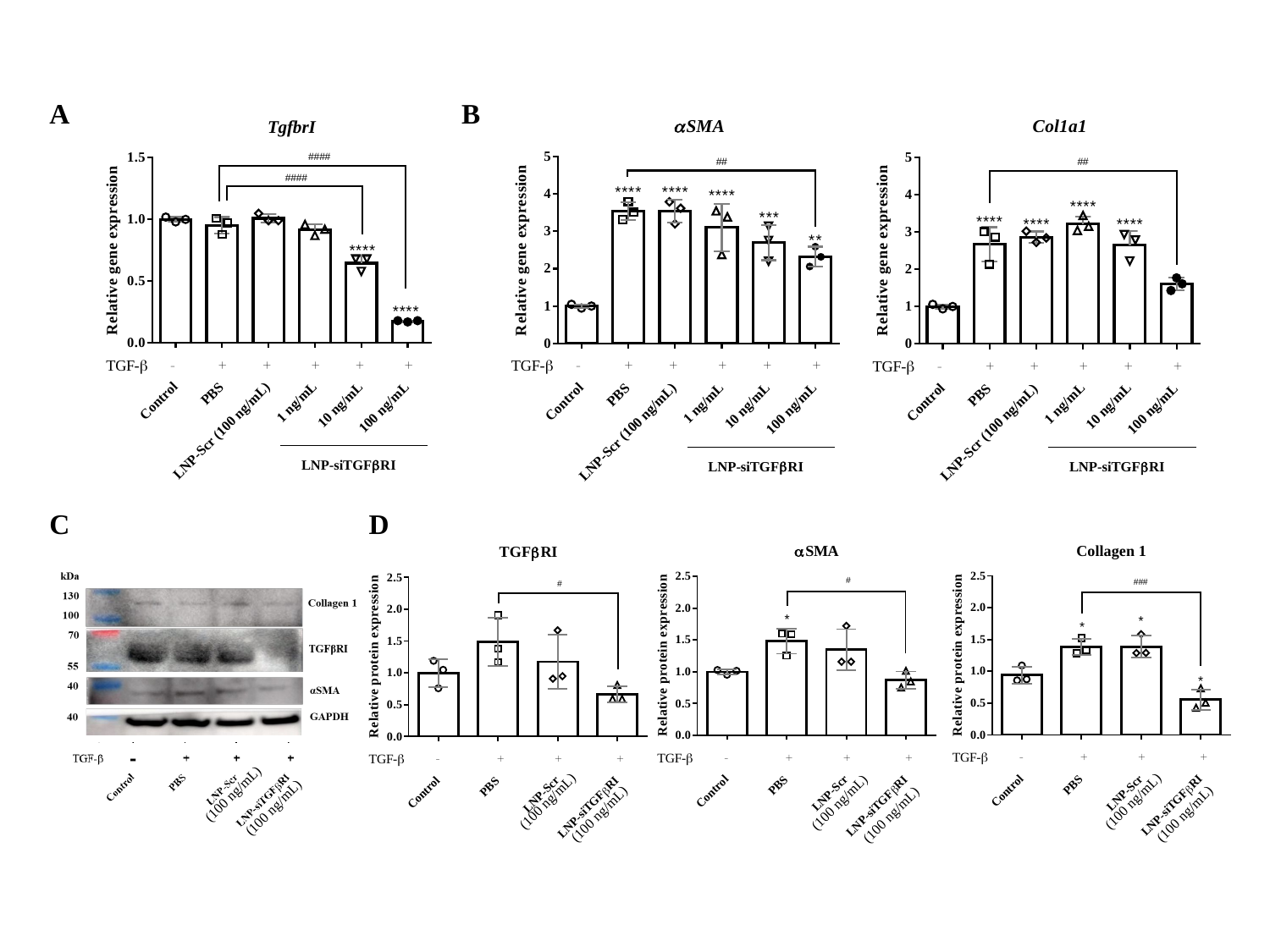

A
B
C
D
(100 ng/mL)
(100 ng/mL)
(100 ng/mL)
(100 ng/mL)
(100 ng/mL)
(100 ng/mL)
(100 ng/mL)
(100 ng/mL)

Supplement: S1 Data — (ZIP) [file pntd.0012502.s004.zip › Raw data/fig.2 WB raw data/Fig. 2D collagen rev_0828.pptx]

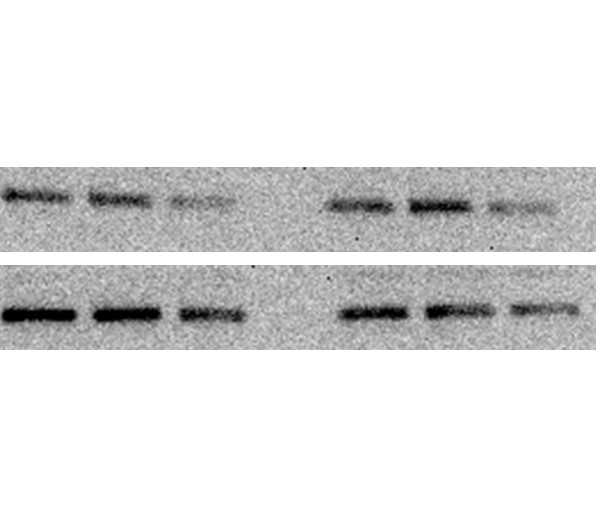

Supplement: S1 Data — (ZIP) [file pntd.0012502.s004.zip › Raw data/fig.2 WB raw data/Fig2 WB aSMA.jpg]

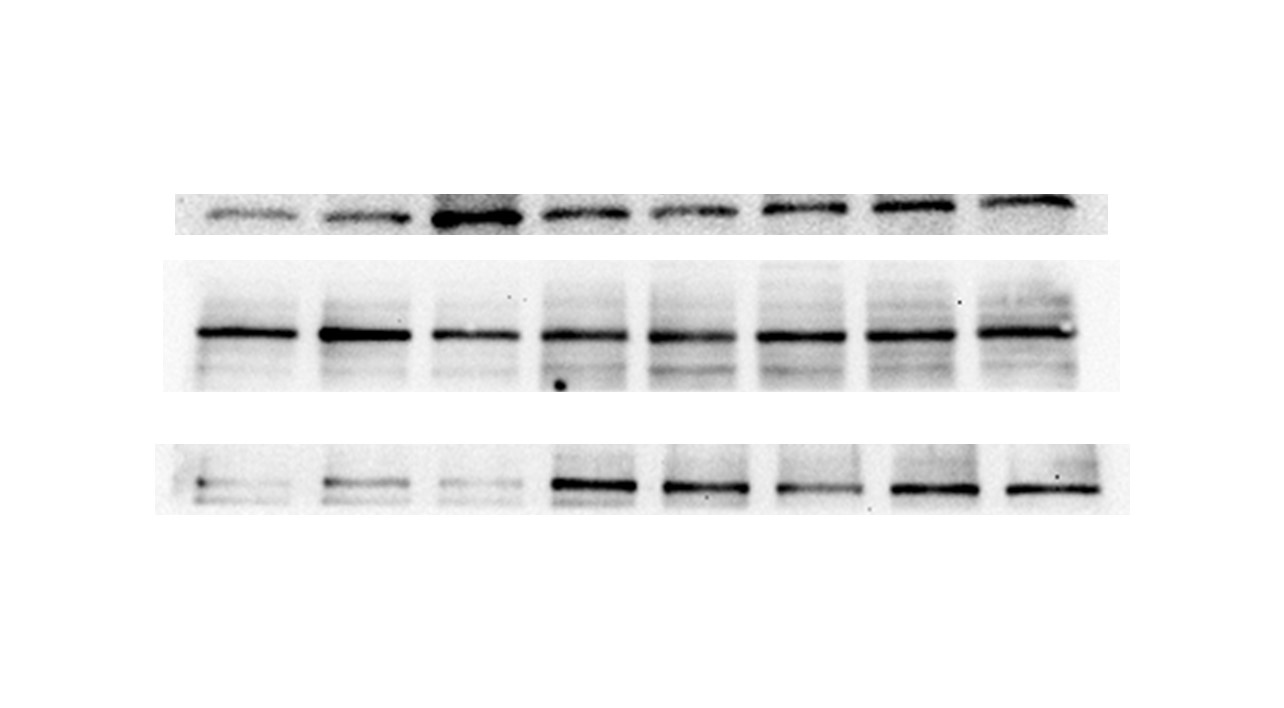

Supplement: S1 Data — (ZIP) [file pntd.0012502.s004.zip › Raw data/fig.2 WB raw data/Fig2 WB Collagen.jpg]

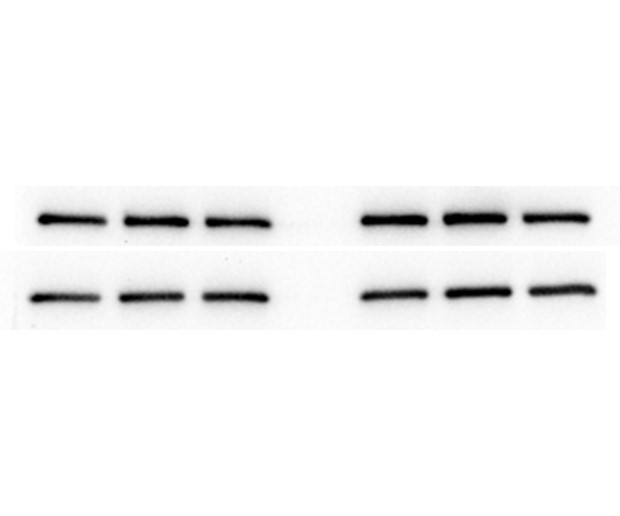

Supplement: S1 Data — (ZIP) [file pntd.0012502.s004.zip › Raw data/fig.2 WB raw data/Fig2 WB GAPDH.jpg]

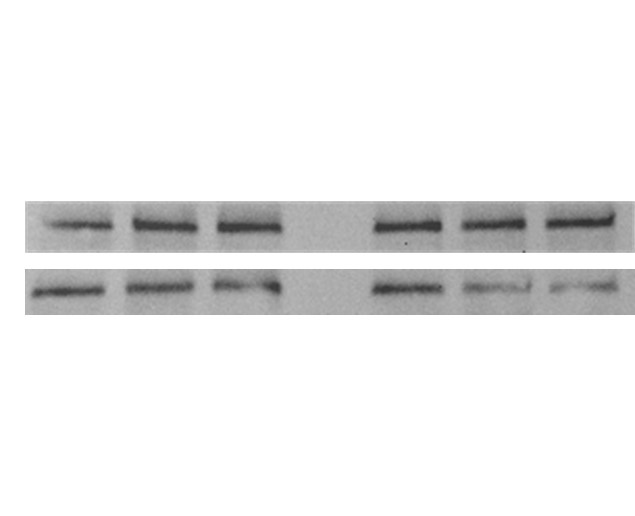

Supplement: S1 Data — (ZIP) [file pntd.0012502.s004.zip › Raw data/fig.2 WB raw data/Fig2 WB TGFbRI.jpg]

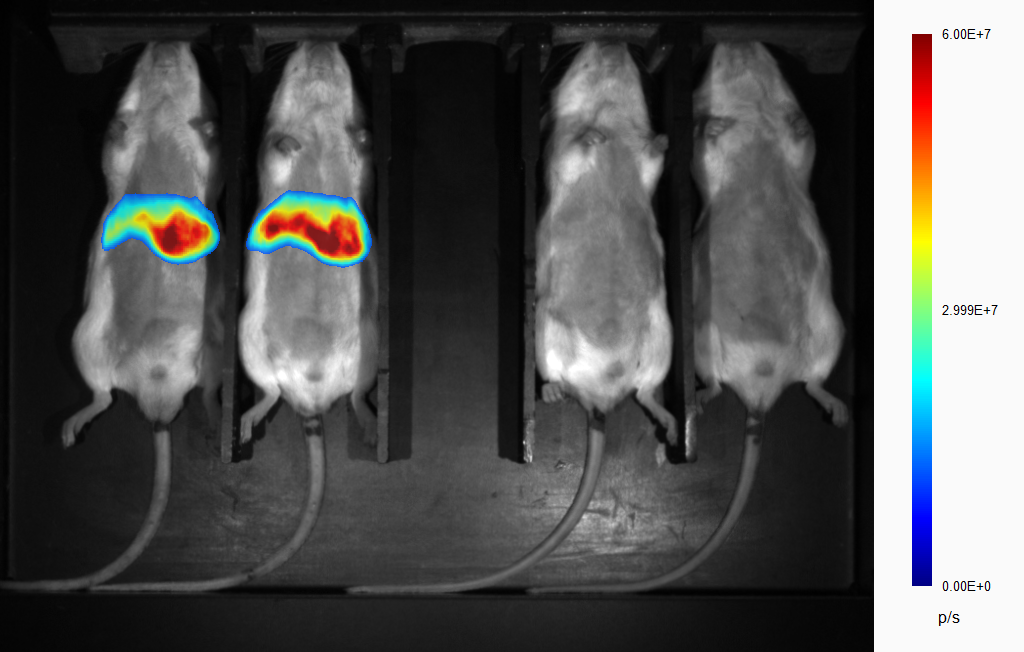

Supplement: S1 Data — (ZIP) [file pntd.0012502.s004.zip › Raw data/fig.4 raw images/Fig 4A-BioD.png]

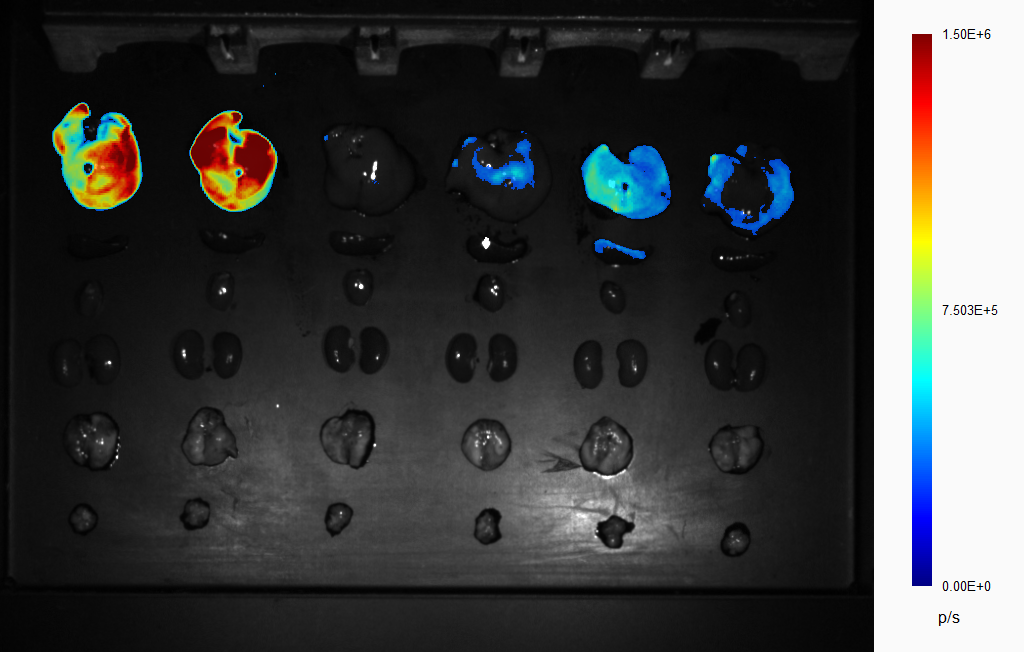

Supplement: S1 Data — (ZIP) [file pntd.0012502.s004.zip › Raw data/fig.4 raw images/Fig 4B-ex vivo.png]

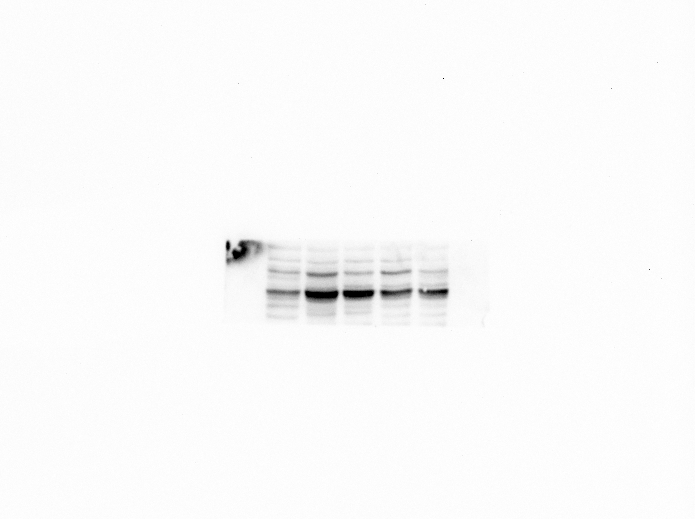

Supplement: S1 Data — (ZIP) [file pntd.0012502.s004.zip › Raw data/fig.5 WB raw data/a-SMA.tif]

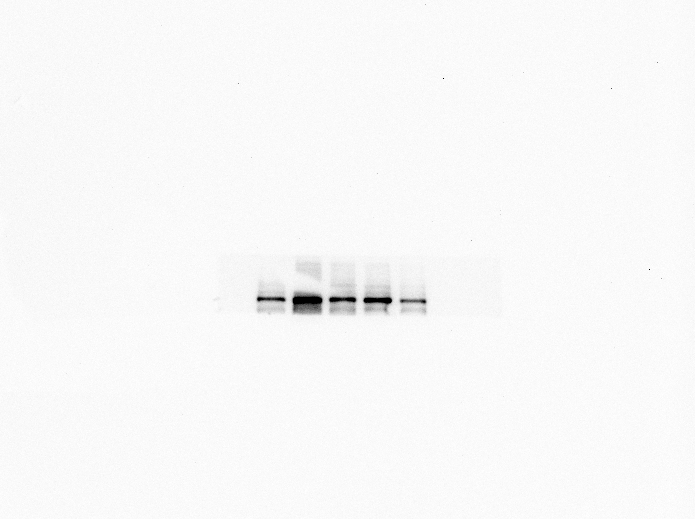

Supplement: S1 Data — (ZIP) [file pntd.0012502.s004.zip › Raw data/fig.5 WB raw data/Collagen.tif]

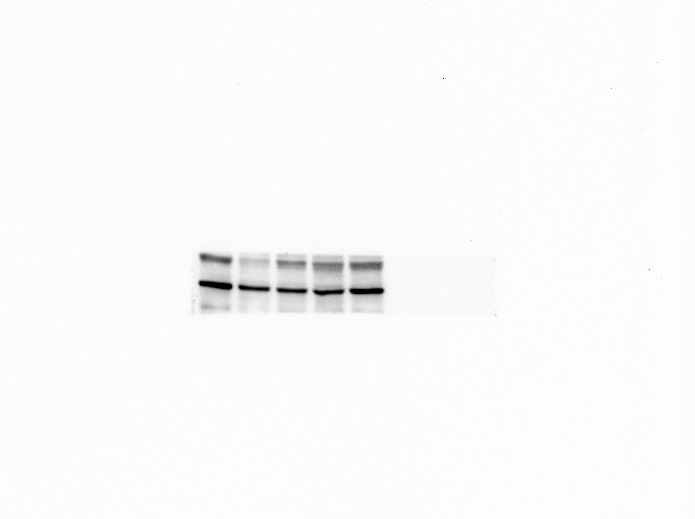

Supplement: S1 Data — (ZIP) [file pntd.0012502.s004.zip › Raw data/fig.5 WB raw data/GADPH.tif]

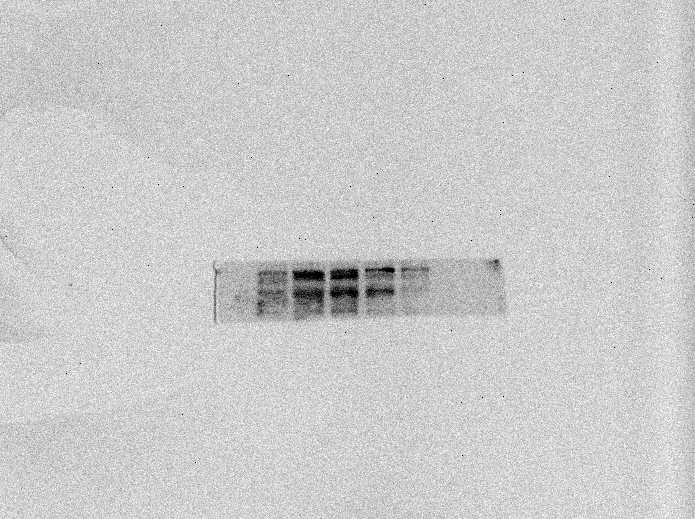

Supplement: S1 Data — (ZIP) [file pntd.0012502.s004.zip › Raw data/fig.5 WB raw data/TGFBR1.tif]
