# Supplementary material for: Development of a Lipid-encapsulated TGFβRI-siRNA Drug for Liver Fibrosis Induced by Schistosoma mansoni
Source: PLoS Negl Trop Dis. 2024 Sep 12;18(9):e0012502. doi: 10.1371/journal.pntd.0012502 (PMC11421824; doi:10.1371/journal.pntd.0012502)
Supplement: S2 Table — * p < 0.05, ** p < 0.01, as compared to PBS positive control group. (PDF) [file pntd.0012502.s002.pdf]

Table S2. The worm recovery burden and reduction rate of each group were calculated in the study.

Groups	Adult worm burden (mean ± SD)	Reduction rate (%)
PBS	44.85 ± 12.30	-
LNP-Scr	18.00 ± 2.88	59.87
0.1 mg/kg LNP-siTGFβRI	7.5 0± 1.01**	83.28
1 mg/kg LNP-siTGFβRI	8.5 0± 2.01*	81.05

* $p < 0.05$, ** $p < 0.01$, as compared to PBS positive control group.
