# Supplementary material for: Development of a Lipid-encapsulated TGFβRI-siRNA Drug for Liver Fibrosis Induced by Schistosoma mansoni
Source: PLoS Negl Trop Dis. 2024 Sep 12;18(9):e0012502. doi: 10.1371/journal.pntd.0012502 (PMC11421824; doi:10.1371/journal.pntd.0012502)
Supplement: S3 Table — Arrows in table represent the direction of cell frequency change in infected animals in comparison to naïve. * p < 0.05, ** p < 0.01, *** p < 0.001, **** p < 0.0001, ns = non-significant (p > 0.05) compared with naïve group. (PDF) [file pntd.0012502.s003.pdf]

Table S3. Statistics of immune cell frequencies of treatments and their comparison to naïve mice. Arrows in table represent the direction of cell frequency change in infected animals in comparison to naïve. * p < 0.05, ** p < 0.01, *** p < 0.001, **** p < 0.0001, ns = non-significant (p > 0.05) compared with naïve group.

Cell type	Treatment		
	PBS	low dose	High dose
**Total B cells**	↓****	↓*	↑***
**Total T cells**	↓****	↑**	ns
Th	↓***	↑***	ns
Active (MHC Class II+) Th	↑***	ns	ns
Active (CD44+) Th	↑*	ns	ns
Naïve (CD62L+) Th	↓****	↓****	↓****
Th1	ns	↑****	↑*
Th2	↑*	ns	ns
Th17	ns	ns	ns
Th22	ns	ns	ns
Tfh	↓****	ns	ns
Memory (CD62L-MHC Class II-) Th	ns	↑****	↑****
Regulatory Th	ns	↑****	↑****
Tc	ns	ns	↓*
Active Tc	↑**	ns	ns
Active (CD44+) Tc	ns	ns	ns
Naïve (CD62L+) Tc	ns	↓*	↓*
Tc1	↑**	ns	ns
Tc2	↓***	↓***	↓***
Tc17	↓****	↓****	↓****
Tc22	ns	ns	ns
Tfc	↓***	↓****	↓****
Memory (CD62L-MHC Class II-) Tc	↑*	ns	ns
Regulatory Tc	ns	ns	ns
**Total NK cells**	↑****	ns	↓***
CD56b NK	ns	ns	ns
CD56d NK	↑****	ns	ns
CD56b	ns	ns	ns
CD56d	↑****	ns	↓*
**Total NKT cells**	ns	ns	ns
**Total DCs**	ns	ns	ns
**Total Monocyte**	ns	ns	ns
